# Supplementary material for: Quadrupolar 23Na+ NMR relaxation as a probe of subpicosecond collective dynamics in aqueous electrolyte solutions
Source: Nat Commun. 2023 Jan 5;14:84. doi: 10.1038/s41467-022-35695-3 (PMC9816157; doi:10.1038/s41467-022-35695-3)
Supplement: Supplementary file 1 — Supplementary Information [file 41467_2022_35695_MOESM1_ESM.pdf]

## Supplementary Information

# Quadrupolar $^{23}Na^{+}$ NMR Relaxation as a Probe of Subpicosecond Collective Dynamics in Aqueous Electrolyte Solutions

Iurii Chubak,[1,*] Leeor Alon,[2,3,*] Emilia V. Silletta,[4,5]
Guillaume Madelin,[2,3] Alexej Jerschow,[6,†] and Benjamin Rotenberg[1,‡]

[1] *Sorbonne Université CNRS, Physico-Chimie des électrolytes et Nanosystèmes Interfaciaux, F-75005 Paris, France*
[2] *New York University School of Medicine, Department of Radiology, Center for Biomedical Imaging, 660 First Avenue, New York, NY 10016, USA*
[3] *Center for Advanced Imaging Innovation and Research, Department of Radiology, New York University Grossman School of Medicine, New York, NY, 10016, USA*
[4] *Universidad Nacional de Córdoba, Facultad de Matemática, Astronomía, Física y Computación, Medina Allende s/n, X5000HUA, Córdoba, Argentina.*
[5] *Instituto de Física Enrique Gaviola, CONICET, Medina Allende s/n, X5000HUA, Córdoba, Argentina.*
[6] *New York University, Department of Chemistry, 100 Washington Square E, New York, NY 10003, USA*

### CONTENTS

Supplementary Note 1: Theory 1

Supplementary Note 2: Force field parameters 2

Supplementary Note 3: Impact of dipole-dipole couplings 3

Supplementary Note 4: Finite-size effects 4

Supplementary Note 5: Structure of NaCl solutions 4

Supplementary Note 6: Effect of the local solvation shell structure on the effective Sternheimer factor 8

Supplementary Note 7: Effect of the local solvation shell structure on the electric field gradient variance 8

Supplementary Note 8: Solvation shell resolved electric field gradient relaxation 9

Supplementary Note 9: Fitting of electric field gradient relaxation functions 11

Supplementary Note 10: Quadrupolar coupling constant 21

Supplementary Note 11: Water dipole reorientation 21

Supplementary Note 12: Quadrupolar relaxation within a Stokes-Einstein-Debye model 21

Supplementary Note 13: Quadrupolar relaxation of $^{23}Na^{+}$ in other electrolyte solutions 21

Supplementary References 24

### Supplementary Note 1: Theory

In this section, we provide a more detailed derivation of the expressions used for calculating the quadrupolar relaxation rates in Section II A of the main text. As given by the formalism of Spiess et al. [1–3], the longitudinal $1/T_1$ and transverse $1/T_2$ quadrupolar relaxation rates of nuclei with spin $I > 1/2$ can be determined through

$$\frac{1}{T_1(\omega_0)} = \frac{2I+3}{40I^2(2I-1)}\left(\frac{eQ}{\hbar}\right)^2 G^Q_{2,0}(\omega_0) \quad (1)$$

$$\frac{1}{T_2(\omega_0)} = \frac{2I+3}{40I^2(2I-1)}\left(\frac{eQ}{\hbar}\right)^2 G^Q_{2,\pm1}(\omega_0) \quad (2)$$

where $\hbar$ is the reduced Planck constant, $eQ$ is the quadrupolar moment of the nucleus, and $\omega_0 = \gamma B_0$ is its Larmor frequency of the nucleus in the external magnetic field $B_0$ with $\gamma$ denoting its gyromagnetic ratio. $G^Q_{2,0}(\omega)$ and $G^Q_{2,\pm1}(\omega)$ are spectral densities of the EFG at the nucleus position (see below). As the NMR relaxation of spin components may generally not be single-exponential [4], strictly speaking, the longitudinal and transverse quadrupolar relaxation rates above can be only defined under the following conditions [1]: (*i*) for the nuclear spin $I = 1$; (*ii*) provided that the spin subsystem can be described by a sufficiently high spin-temperature; (*iii*) in the extreme narrowing regime, for which the typical microscopic EFG correlation time $\tau_c$ is much smaller in comparison to the Larmor period $\omega_0^{-1}$ of the nucleus, yielding $\omega_0\tau_c \ll 1$. As we discuss in the main text, the latter condition is in fact fulfilled for $^{23}Na$ in aqueous solutions considered in this work and implies that the

* Equal contribution
† alexej.jerschow@nyu.edu
‡ benjamin.rotenberg@sorbonne-universite.fr

spectral densities in Eqs. (1) and (2) can be effectively evaluated at $\omega = 0$.

The EFG spectral densities $G^Q_{2,0}(\omega)$ and $G^Q_{2,\pm1}(\omega)$ can be explicitly written as [1]:

$$G^Q_{2,0}(\omega) = 4g^Q_{2,2}(2\omega) + 4g^Q_{2,-2}(-2\omega) + g^Q_{2,1}(\omega) + g^Q_{2,-1}(-\omega) \tag{3}$$

$$G^Q_{2,\pm1}(\omega) = 2g^Q_{2,\mp2}(\mp2\omega) + 3g^Q_{2,\mp1}(\mp\omega) + 2g^Q_{2,\pm1}(\pm\omega) + 3g^Q_{2,0}(0), \tag{4}$$

where $g^Q_{l,m}$ are half-sided Fourier transforms of the autocorrelation functions of the spherical EFG components $V_{l,m}$ ($l = 2$, $m = 0, \pm1, \pm2$):

$$g_{l,m}(\omega) = \int_0^\infty \mathrm{d}t\, e^{i\omega t} \left\langle V_{l,m}(t) V^*_{l,m}(0) \right\rangle \tag{5}$$

with the brackets $\langle \dots \rangle$ denoting the time averaging and $V_{l,m}$ for $l = 2$ given by [3]

$$V_{2,0} = \sqrt{\frac{3}{2}} V_{zz} \tag{6}$$

$$V_{2,\pm1} = \mp V_{xz} - iV_{yz} \tag{7}$$

$$V_{2,\pm2} = \frac{1}{2}\left(V_{xx} - V_{yy}\right) \pm iV_{xy}, \tag{8}$$

where $V_{\alpha\beta}$ ($\alpha, \beta = x, y, z$) stand for the Cartesian components of the EFG tensor at the nucleus.

The rotational invariance of the considered system implies that all autocorrelation functions $\langle V_{l,m}(t) V^*_{l,m}(0)\rangle$ in Eq. (5) for different $m$ become equal [1]. Combined with the extreme narrowing of the signal, we find from Eqs. (3) and (4) that $G^Q_{2,0}(0) = G^Q_{2,\pm1}(0)$ and can be recast as follows:

$$G^Q_{2,0}(0) = 2\int_0^\infty \mathrm{d}t \left\langle \mathbf{V}(t){:}\mathbf{V}(0) \right\rangle, \tag{9}$$

where $\langle \mathbf{V}(t){:}\mathbf{V}(0)\rangle = \sum_{\alpha,\beta} \langle V_{\alpha\beta}(t) V_{\alpha\beta}(0)\rangle$ with $\alpha, \beta = x, y, z$. Accordingly, the two effective quadrupolar rates $1/T_1$ and $1/T_2$ also equalize and are given by

$$\frac{1}{T_1} = \frac{2I+3}{20I^2(2I-1)} \left(\frac{eQ}{\hbar}\right)^2 \int_0^\infty \mathrm{d}t \left\langle \mathbf{V}(t){:}\mathbf{V}(0) \right\rangle \tag{10}$$

The Eq. (10) above can be rewritten in terms of the variance of the EFG tensor $\langle \mathbf{V}^2 \rangle \equiv \langle \mathbf{V}(0){:}\mathbf{V}(0)\rangle$ and the effective EFG correlation time $\tau_c$:

$$\tau_c = \left\langle \mathbf{V}^2 \right\rangle^{-1} \int_0^\infty \mathrm{d}t \left\langle \mathbf{V}(t){:}\mathbf{V}(0) \right\rangle, \tag{11}$$

yielding the final expression

$$\frac{1}{T_1} = \frac{2I+3}{20I^2(2I-1)} \left(\frac{eQ}{\hbar}\right)^2 \left\langle \mathbf{V}^2 \right\rangle \tau_c \tag{12}$$

Similarly to the intramolecular quadrupolar relaxation, the value of $\langle \mathbf{V}^2 \rangle$ can be recast in terms of the so-called quadrupolar coupling constant (QCC) $C_Q$ [7]. The latter is defined through the the three eigenvalues of the instantaneous EFG tensor at the ion position, $V_{33}$, $V_{22}$, and $V_{11}$, that are ordered in a descending manner, $|V_{33}| \geq |V_{22}| \geq |V_{11}|$. It can be shown that the ensemble average $\langle \mathbf{V}^2 \rangle$ satisfies

$$\left\langle \mathbf{V}^2 \right\rangle = \frac{3}{2}\left[\left\langle V_{33}^2 \right\rangle + \frac{1}{3}\left\langle (V_{22} - V_{11})^2 \right\rangle\right] \tag{13}$$

permitting us to define the value $(eq)^2 \equiv \langle V_{33}^2 \rangle$ and the asymmetry parameter $\eta_Q \equiv \left\langle (V_{22} - V_{11})^2 \right\rangle / \left\langle V_{33}^2 \right\rangle$. Finally, the QCC can be defined as

$$C_Q = \left(\frac{e^2 Qq}{\hbar}\right)\sqrt{1 + \frac{\eta_Q^2}{3}} \equiv \sqrt{\frac{2\langle \mathbf{V}^2 \rangle}{3}} \left(\frac{eQ}{\hbar}\right), \tag{14}$$

which enables to rewrite Eq. (12) as follows:

$$\frac{1}{T_1} = \frac{3}{40} \frac{2I+3}{I^2(2I-1)} C_Q^2 \tau_c \tag{15}$$

It should be noted that for a purely intramolecular mechanism the quadrupolar relaxation is primarily driven by the molecule reorientation [7]. In such case, the components of the EFG tensor are fixed in a reference frame associated with the molecule that makes the values of $eq$ and $\eta_Q$ discussed above constant. On the other hand, for an intermolecular mechanism, as for monoatomic quadrupolar ions considered here, the components of the EFG tensor at the nucleus position are not fixed in any reference frame, but are stochastic quantities, typically satisfying a Gaussian distribution [8]. Precisely because of the latter fact, in the present case $eq$ and $\eta_Q$ are determined from the statistically averaged values of the eigenvalues.

### Supplementary Note 2: Force field parameters

As mentioned in the main text, aqueous NaCl solutions were simulated using the recently-developed Madrid-2019 [5, 6] FF for aqueous electrolytes that employs scaled ionic charges. The electrolyte FF is based on the TIP4P/2005 water model [9]. The interparticle interaction energy $V(r_{ij})$ in the FF is given by a sum of Coulomb and Lennard-Jones (LJ) potentials:

$$V(r_{ij}) = \frac{q_i q_j}{4\pi\epsilon_0 r_{ij}} + 4\epsilon_{ij}\left[\left(\frac{\sigma_{ij}}{r_{ij}}\right)^{12} - \left(\frac{\sigma_{ij}}{r_{ij}}\right)^{6}\right], \tag{16}$$

where $q_i$ and $q_j$ the charges of the $i^{\mathrm{th}}$ and $j^{\mathrm{th}}$ atoms, $r_{ij}$ is the distance between two particles, $\epsilon_0$ is the vacuum permittivity, $\epsilon_{ij}$ is the minimum of the LJ potential, and $\sigma_{ij}$ is the LJ diameter. In the TIP4P/2005 model [9], the rigid water molecules are comprised of four interaction sites: an oxygen atom with $q_\mathrm{O} = 0$ that is used as a LJ interaction center, two hydrogens with $q_\mathrm{H} = 0.5564e$,

Supplementary Table 1. LJ interaction parameters of the Madrid-2019 FF for aqueous NaCl, NaBr, and NaF solutions [5, 6]

| **NaCl** | Particle pair | | | | | |
|---|---|---|---|---|---|---|
| | Na–Na | Na–Cl | Na–O | Cl–Cl | Cl–O | O–O |
| $\sigma_{ij}$ (Å) | 2.21737 | 3.00512 | 2.60838 | 4.69906 | 4.23867 | 3.15890 |
| $\epsilon_{ij}$ (kJ/mol) | 1.472356 | 1.438894 | 0.793388 | 0.076923 | 0.061983 | 0.774908 |
| **NaBr** | Na–Na | Na–Br | Na–O | Br–Br | Br–O | O–O |
| $\sigma_{ij}$ (Å) | 2.21737 | 3.38500 | 2.60838 | 4.82525 | 4.19850 | 3.15890 |
| $\epsilon_{ij}$ (kJ/mol) | 1.472356 | 0.35677 | 0.793388 | 0.112795 | 0.1000 | 0.774908 |
| **NaF** | Na–Na | Na–F | Na–O | F–F | F–O | O–O |
| $\sigma_{ij}$ (Å) | 2.21737 | 3.003595 | 2.60838 | 3.78982 | 3.77450 | 3.15890 |
| $\epsilon_{ij}$ (kJ/mol) | 1.472356 | 0.2135172 | 0.793388 | 0.0309637 | 0.1000 | 0.774908 |

and a massless M-site with $q_{\mathrm{M}} = -2q_{\mathrm{H}}$ that is located along the H-O-H bisector direction. The H-O-H angle is 104.52°, the O-H distance is 0.9572 Å, and the O-M distance is 0.1546 Å. For aqueous NaCl solutions, explicit values of the LJ parameters are listed in Tab. 1. The charges of the $Na^+$ cations and $Cl^-$ anions are $q_{\mathrm{Na}} = +0.85e$ and $q_{\mathrm{Cl}} = -0.85e$, respectively, with $e$ denoting the fundamental unit of charge.

The use of scaled ionic charges in the Madrid-2019 FF [5, 6] enables not only an excellent description of the solution densities, but also a good representation of the dynamic properties of electrolytes, as given by the solution viscosity, self-diffusion coefficients of ions and water, as well as the quadrupolar NMR relaxation rates for a series of ions at infinite dilution [10]. As we show in Fig. 1, the NaCl densities obtained explicitly in $NPT$ simulations at $P = 1$ bar are in excellent agreement with the experimental values within the considered range of temperatures and concentrations [11]. To compute equilibrium NaCl densities in the Madrid-2019 FF, we performed $NPT$ simulations of length 1 ns using the MetalWalls open-source code [12] by coupling the systems to chains of Nosé-Hoover barostats and thermostats [13–15], both with a time constant of 1 ps. Finally, at a given system temperature $T$ and salt concentration $c$, NaCl solutions were initialized and equilibrated at the resulting solution densities $\rho(c, T)$ that corresponds to the 1 bar isobar and the production runs were performed in the $NVT$ ensemble. In each case, $N_{\mathrm{w}} = 1000$ water molecules and $N_{\mathrm{p}}$ ion pairs were simulated in a box at a given density $\rho(c, T)$ (precise parameters are listed in Tab. 2).

In addition to the NaCl systems, we have also simulated aqueous NaBr and NaF solutions using the extended Madrid-2019 FF parameters [6] (listed in Tab. 1) using the same protocol as described above. NaBr solutions were simulated at the same salt concentrations as in the NaCl case, whereas NaF salts were simulated at $c = 0.06, 0.5$, and 1 m because of their rather small solubility in water [6]. The simulations were performed at $T = 25$ °C using equilibrium densities obtained in $NPT$ simulations at 1 bar. The latter solution densities are also in excellent agreement with the experimental ones within the considered range of salt concentrations [6].

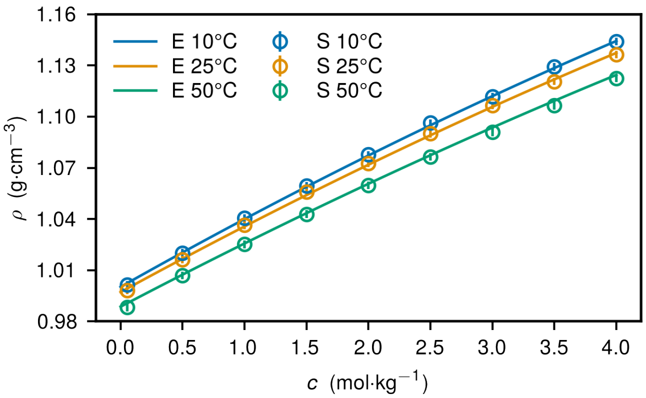


**Supplementary Figure 1**. Density of aqueous NaCl solutions as a function of the salt concentration $c$ in experiments (solid lines) and simulations in the $NPT$ ensemble (open circles) at $P = 1$ bar and different temperatures. The standard error from multiple independent simulation runs is smaller than the symbol size.

Supplementary Table 2. Number of ion pairs $N_{\mathrm{p}}$ and respective salt concentrations $c$ in the simulation boxes with aqueous NaCl solutions. In each case, the system contained $N_{\mathrm{w}} = 1000$ water molecules.

| $N_{\mathrm{p}}$ | 1 | 9 | 18 | 27 | 36 | 45 | 54 | 63 | 72 |
|---|---|---|---|---|---|---|---|---|---|
| c (mol·kg$^{-1}$) | 0.06 | 0.5 | 1.0 | 1.5 | 2.0 | 2.5 | 3.0 | 3.5 | 4.0 |

### Supplementary Note 3: Impact of dipole-dipole couplings

In this section, we will estimate a potential effect of the dipole-dipole coupling mechanism on the longitudinal relaxation rate $T_1^{-1}$ of $^{23}Na^+$ ions. Besides the quadrupolar contribution to the rate discussed in Sec. 1 that is

Supplementary Table 3. Estimates for the contribution $T_{1X}^{-1}$ to the longitudinal rate of $^{23}Na^{+}$ that arises from the dipole-dipole interaction between the spin of the sodium ion and the spin $I_X$ of $X = {}^{1}H$, $^{23}Na^{+}$, and $^{35}Cl^{-}$. $\gamma_X$ are the respective nuclear gyro-magnetic ratios and $r$ is the location of the first peak of Na–$X$ radial distribution functions (see Fig. 3). In all cases, we assumed that $\tau_c^{dd} \approx 4$ ps.

| $X$ | $I_X$ | $\frac{\gamma_X}{2\pi}$ (MHz·T$^{-1}$) | $r$ (Å) | $T_{1X}^{-1}$ (s$^{-1}$) |
|---|---|---|---|---|
| $^{1}H$ | $\frac{1}{2}$ | 42.577 | 2.89 | $2.7{\cdot}10^{-4}$ |
| $^{23}Na^{+}$ | $\frac{3}{2}$ | 11.262 | 3.64 | $3.6{\cdot}10^{-5}$ |
| $^{35}Cl^{-}$ | $\frac{3}{2}$ | 4.176 | 2.86 | $2.6{\cdot}10^{-5}$ |

expected to dominate the NMR relaxation for quadrupolar nuclei [7], additional additive contributions $T_{1X}^{-1}$ to $T_1^{-1}$ might arise from the $^{23}Na^{+}$–$X$ dipole-dipole spin interaction, where $X = {}^{1}H$, $^{23}Na^{+}$, and $^{35}Cl^{-}$. In this case, the NMR relaxation for two unlike spins $I$ and $I_X$ is determined from the dipole-dipole ACF $G(t)$ [16, 17]:

$$G(t) = \frac{1}{4}\left(\frac{\mu_0}{4\pi}\right)^2 \hbar^2\gamma_I^2\gamma_X^2 I_X(I_X+1)\times \left\langle \left[\frac{3\cos^2\theta(t+\tau)-1}{r^3(t+\tau)}\right]\left[\frac{3\cos^2\theta(\tau)-1}{r^3(\tau)}\right]\right\rangle_\tau , \quad (17)$$

where we assume that the spin $I$ is relaxed by the spin $I_X$, $\gamma_I$ and $\gamma_X$ are the two nuclear gyro-magnetic ratios, $\mu_0$ is the vacuum permeability, $\hbar$ is the Planck constant, $r(t)$ is the distance between two spins at time $t$, $\theta(t)$ is the angle between the distance vector $\mathbf{r}(t)$ and the magnetic field direction at time $t$, and brackets $\langle\cdots\rangle_\tau$ denote ensemble averaging. Provided that the extreme narrowing condition holds, the longitudinal rate contribution $T_{1X}^{-1}$ is given by [16]:

$$\frac{1}{T_{1X}} = \frac{20c_X}{3}\,G(0)\,\tau_c^{dd}, \quad (18)$$

where $G(0)$ is determined from Eq. (17) at $t = 0$, $\tau_c^{dd}$ is the correlation time of the dipole-dipole interaction that is evaluated as the integral of the normalized ACF $G(t)/G(0)$, and the constant $c_X$ is equal to 3/2 in the case of like spins with $I = I_X$ and 1 otherwise [16]. To estimate $G(0)$, let us assume that the angular part in Eq. (17) is uncorrelated to the radial one at $t = 0$, yielding $\langle[3\cos^2\theta(\tau)-1]^2\rangle_\tau = 4/5$ [16]. Thus,

$$G(0) = \frac{1}{5}\left(\frac{\mu_0}{4\pi}\right)^2 \hbar^2\gamma_I^2\gamma_X^2 I_X(I_X+1)\left\langle\frac{1}{r^6}\right\rangle. \quad (19)$$

We estimate the value $\langle 1/r^6\rangle$ from the first peak of the respective Na–$X$ radial distribution functions (see Fig. 3 below) and assume that $\tau_c^{dd} \approx 4$ ps that corresponds to the time scale of intermolecular $^{1}H$ dipole-dipole relaxation in pure water at room temperature conditions [18]. The resulting values are listed in Tab. 3. The potential contribution to the NMR rate coming from the dipole-dipole interaction with $^{1}H$ is the largest ($\approx 2.7{\cdot}10^{-4}$ s$^{-1}$), whereas those with $^{23}Na^{+}$ and $^{35}Cl^{-}$ are much smaller. Nevertheless, the overall dipole-dipole contribution is estimated to be orders of magnitude smaller than the quadrupolar rate ($\approx 16$ s$^{-1}$) that we extract in our MD simulations at low salt concentrations.

### Supplementary Note 4: Finite-size effects

To assess the finite-size effects on the EFG ACFs and the resulting NMR relaxation rates, we have simulated three systems at $c = 4$ m with $N_w = 125, 250$ and 500 water molecules, in addition to the original one with $N_w = 1000$, with 9, 18, 35, and 72 ion pairs, respectively, at $T = 25$ °C. As seen in Fig. 2, the reduction of the system size does not influence the resulting ACF of the EFG at the ion position. Interestingly, neither we find any effect on the long-time tail of the ACF that is consistent with a hydrodynamic power law decay $\sim t^{5/2}$ [19]. The emergence of such algebraic decays is typically associated with a superposition of an infinite number of relaxation modes with wave vector $k$ in a liquid [20]. In simulations with a box of size $L$ and periodic boundary conditions, however, $k$ is limited by the minimal value $2\pi/L$, and infinitely small wave numbers $k \to 0$ are not accessible. This causes an apparent exponential relaxation of the ACFs at long times that would otherwise be algebraic in a macroscopic system [20]. Nevertheless, within the considered time scales, we do not observe this effect on the EFG ACFs.

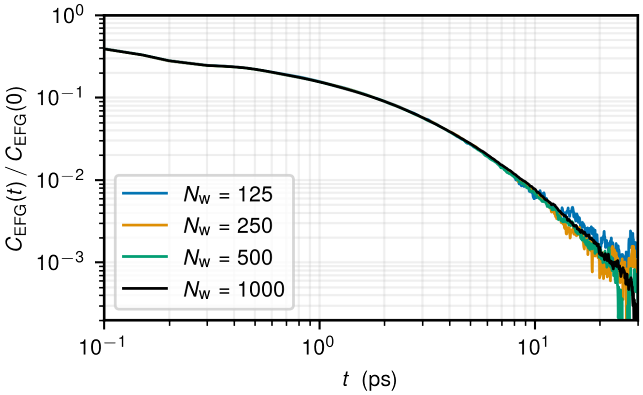


**Supplementary Figure 2**. EFG ACFs at the position of $Na^{+}$ ions for systems with a different number of water molecules $N_w$ at $c = 4$ m and $T = 25$ °C.

### Supplementary Note 5: Structure of NaCl solutions

To elucidate structural changes in aqueous NaCl solutions that arise with changing macroscopic parame-

ters $c$ and $T$ in the Madrid-2019 FF, in Figs. 3 and 4 we show various radial distribution functions (RDFs) for $T = 25$ °C for increasing the salt concentration $c$ and at $c = 1$ m for increasing $T$, respectively. We characterize the solvation shell structure of $Na^+$ ion by means of the Na–O $g_{\mathrm{Na-O}}(r)$ (Figs. 3**a** and 4**a**), Na–H $g_{\mathrm{Na-H}}(r)$ (Figs. 3**b** and 4**b**), Na–Cl $g_{\mathrm{Na-Cl}}(r)$ (Figs. 3**c** and 4**c**), and Na–Na $g_{\mathrm{Na-Na}}(r)$ (Figs. 3**d** and 4**d**) RDFs. For completeness, we also show the Cl–O $g_{\mathrm{Cl-O}}(r)$ (Figs. 3**d** and 4**d**) as well as Cl–Cl $g_{\mathrm{Cl-Cl}}(r)$ (Figs. 3**e** and 4**e**) RDFs. We define the boundaries of the first and the second solvation shells of $Na^+$ from the first two minima of the Na–O RDF, $g_{\mathrm{Na-O}}(r)$ (Figs. 3**a** and 4**a**). Despite a small drift of the first minimum position from around 3.15 Å to 3.19 Å in the range from $c \approx 0.06$ m to $c = 4$ m, for the sake of simplicity we have considered the boundary of the first solvation shell to be at an intermediate value $r_{1s} = 3.17$ Å in all cases. The boundary of the second solvation shell was considered to be at $r_{2s} = 5.39$ Å.

The coordination numbers were obtained as

$$N_{\mathrm{c}} = 4\pi n \int_0^{r_{\mathrm{c}}} \mathrm{d}r\, r^2 g(r), \tag{20}$$

where $g(r)$ is the relevant RDF, $n$ is the number density, and $r_{\mathrm{c}}$ defines the integration limit. For instance, for the oxygen coordination number in the first solvation shell, $g(r) = g_{\mathrm{Na-O}}(r)$, $n$ is the number density of oxygen atoms in the simulation box, and $r_{\mathrm{c}} = r_{1s}$. For the first solvation shell of $Na^+$ at $T = 25$ °C, we find that the oxygen coordination number decreases from around 5.55 to 5.46 for increasing $c$ from 0.06 to 4 m. Concurrently, we find that the hydrogen coordination number decreases from 9.70 to 9.59. Within the two solvation shells of $Na^+$, the number of oxygens decreases from 22.94 to 20.61 for increasing $c$ from 0.06 to 4 m. Naturally, the latter behavior implies that water molecules in the first solvation shell are partly replaced by ions. We find that the latter are mostly due to $Cl^-$ anions, the number of which in the first solvation shell increases from about 0.01 to 0.08. In comparison, the number of $Na^+$ cations in the first solvation shell remains much lower (0.01) even at the highest concentration $c = 4$ m here. Furthermore, we find that at $c = 4$ m there is on average 0.8 cations and 1.9 anions within the second solvation shell of $Na^+$. We find that with increasing $c$, there is an enhanced probability of observing more like-species in the solvation shells of $Na^+$ and $Cl^-$, as evidenced by the increase in the peaks of $g_{\mathrm{Na-Na}}(r)$ and $g_{\mathrm{Cl-Cl}}(r)$ (Figs. 3**d** and 4**f**), whereas an opposite trend is seen for $g_{\mathrm{Na-Cl}}(r)$, whose peaks decrease with increasing $c$. The latter behavior is consistent [21] with the trends found in the MD simulations of aqueous NaCl solutions that employed a deep neural network potential trained on the state-of-the-art DFT calculations using the strongly constrained and appropriately normed (SCAN) functional [22].

The temperature behavior of the RDFs at $c = 1$ m is shown in Fig. 4. For increasing $T$ from 10 °C to 50 °C, the oxygen coordination number in the first solvation shell decreases from 5.55 to 5.45, whereas that of hydrogen from 9.80 to 9.43. Interestingly, we find that first peak of $g_{\mathrm{Na-Cl}}(r)$ at 2.84 Å rises with increasing $T$, corresponding to a small increase of the number of $Cl^-$ anions within the first solvation shell (from around 0.015 at $T = 10$ °C to 0.027 at 50 °C). The latter effect is, however, balanced by the decrease in the height of the second peak of $g_{\mathrm{Na-Cl}}(r)$ at 4.6 Å that leaves the overall number of $Cl^-$ anions within the two solvation shells (0.57) practically unchanged, see Fig. 4**c**.

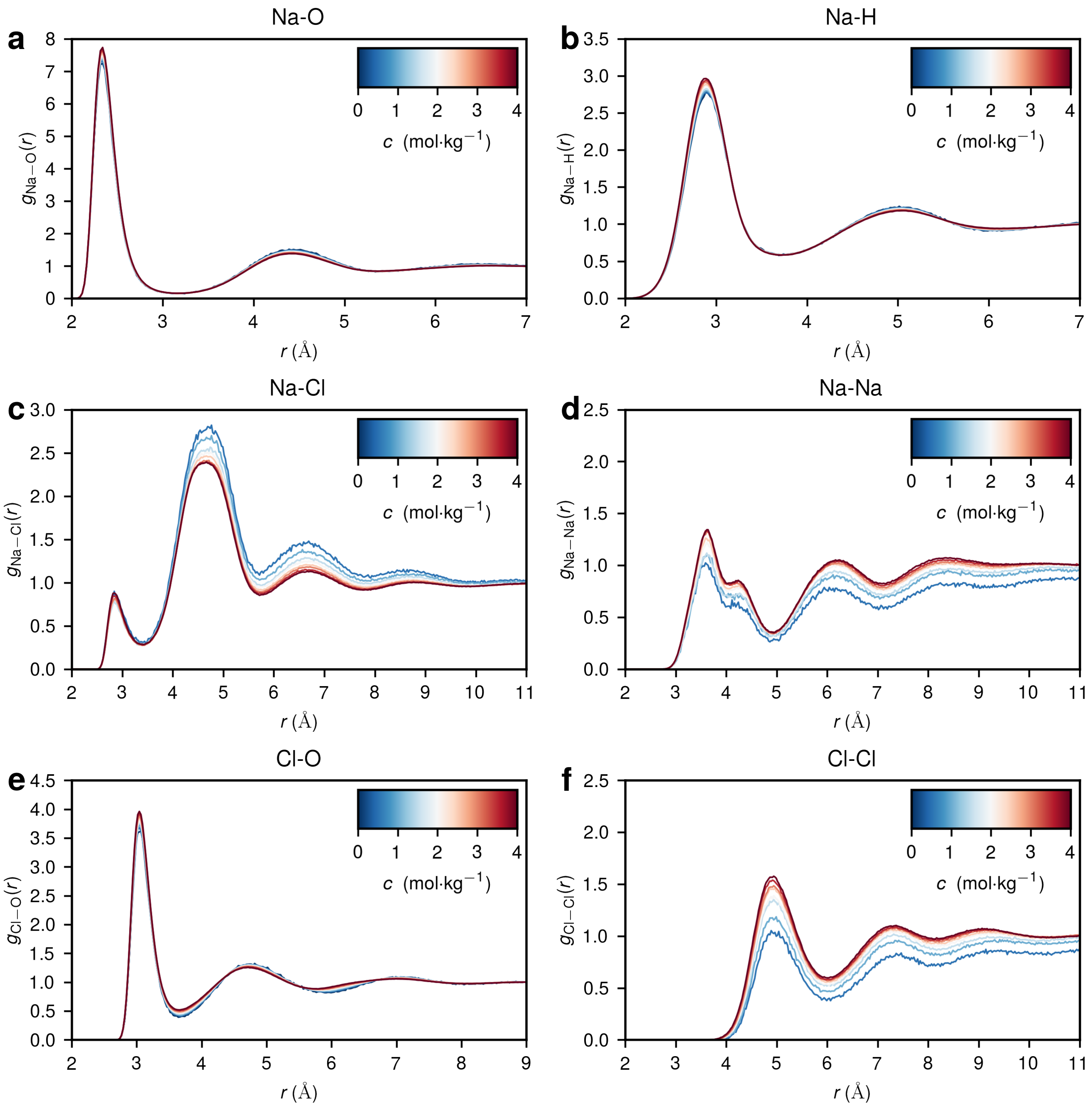


**Supplementary Figure 3**. Structure of NaCl solutions at $T = 25$ °C with increasing the salt concentration $c$. The Na–O **a**, Na–H **b**, Na–Cl **c**, Na–Na **d**, Cl–O **e**, and Cl–Cl **f** RDFs for increasing $c$ in the range from 0.06 to 4 m.

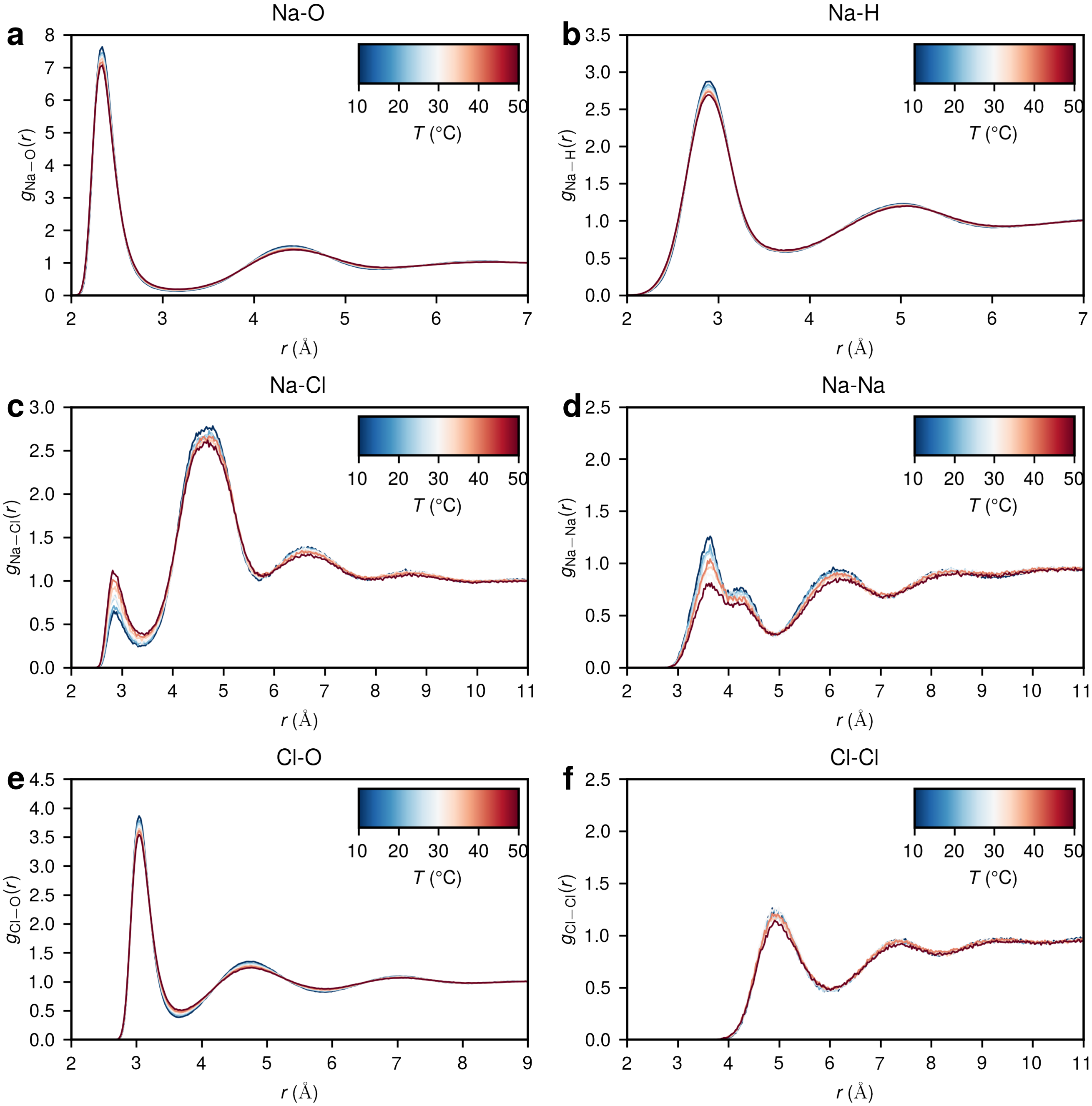


**Supplementary Figure 4**. Structure of NaCl solutions at $c = 1$ m with increasing the system temperature. The Na–O **a**, Na–H **b**, Na–Cl **c**, Na–Na **d**, Cl–O **e**, and Cl–Cl **f** RDFs for increasing $T$ in the range from 10 to 50 °C.

### Supplementary Note 6: Effect of the local solvation shell structure on the effective Sternheimer factor

We classify different cation-anion solvation states by the minima of the Na–Cl RDF, $g_{\mathrm{Na-Cl}}(r)$ (Figs. 3**c** and 4**c**). Given that $r$ denotes the distance between a Na–Cl ion pair, we define the following three possibilities: ($i$) contact ion pairs (CIP) for $r < 3.39$ Å; ($ii$) solvent-shared ion pairs (SIP) $3.39 < r < 5.76$ Å; ($iii$) solvent-separated ion pairs (SSIP) $r > 7.9$ Å. The probability of observing the latter solvation states of $Na^+$ with increasing $c$ at $T = 25$ °C is shown in Fig. 5. We find that SIPs are the most likely states for $c > 1$ m, while there is a rather modest increase in the number of CIPs from few to around ∼10% in the range from 1 to 5 m. The SSIPs are very unlikely at salt concentrations above $c = 3$ m. By integrating $g_{\mathrm{Na-Cl}}(r)$ up to 3.39 Å, we find the number of CIPs to increase from around 0.01 at 0.5 m to 0.1 at 4 m at $T = 25$ °C. In comparison to neural network MD based on the SCAN DFT calculations [21], we find that the computed number of CIPs is ∼2–3 times smaller. The latter is due to the fact that in our case the first peak of $g_{\mathrm{Na-Cl}}(r)$ is much less pronounced than the second one (Figs. 3**c** and 4**c**), as compared to $g_{\mathrm{Na-Cl}}(r)$ reported in Ref. [21].

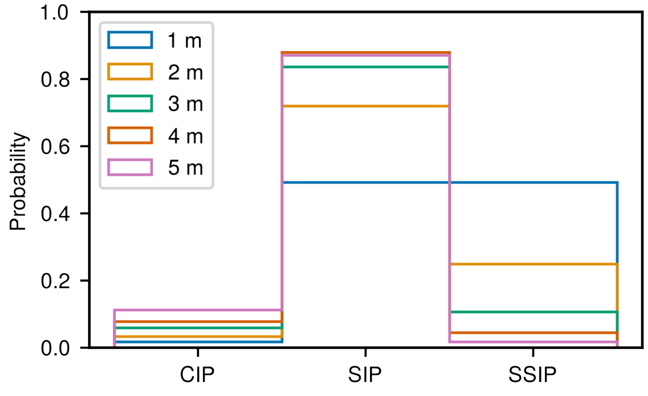


**Supplementary Figure 5**. The likelihood of different $Na^+$ solvation states with increasing the salt concentration $c$ in aqueous NaCl solutions. Probability of observing a contact Na–Cl ion pair (CIP), solvent-shared ion pair (SIP), and solvent-separated ion pair (SSIP) for different salt concentrations indicated in the legend at $T = 25$ °C. At $c = 1$ m (blue line), we find a small difference between the likelihood of finding SIPs and SSIPs that is barely observable in the plotted curve.

In Fig. 6, we show the effective Sternheimer factors extracted on sodium states forming a CIP, SIP, and SSIP with the $Cl^-$ anion. The configuration-resolved $\gamma_{\mathrm{eff}}$ were extracted from a set of at least 50 independent hydration shell configurations of $Na^+$ ions. We find that the ensemble-averaged values of the Sternheimer factor $\gamma_{\mathrm{eff}}$ are consistent with those calculated from a subset of configurations forming the SIP, highlighted with red diamonds in Fig. 6, which constitutes the most probable solvation state of $Na^+$ cations for $c > 1$ mol·kg$^{-1}$ (Fig. 5). The values of Sternheimer factors obtained on a subset of CIPs are somewhat enhanced in comparison to the ensemble average (green triangles in Fig. 6), whereas the ones found for SSIPs, are consistent with the infinite dilution value of $\gamma_{\mathrm{eff}}$ (violet pentagons in Fig. 6).

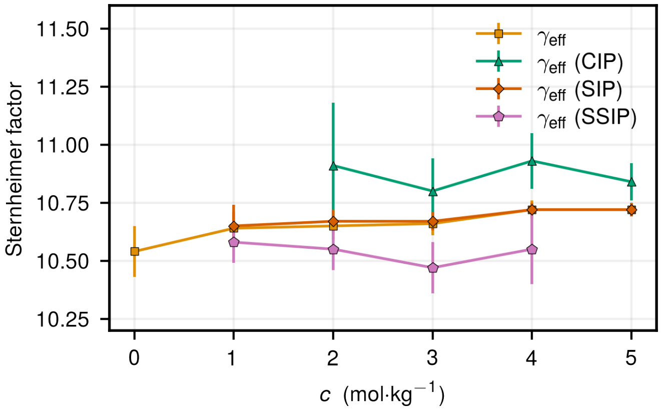


**Supplementary Figure 6**. Configuration-resolved $\gamma_{\mathrm{eff}}$ for $Na^+$ forming contact ion pairs (CIP, green triangles), solvent-shared ion pairs (SIP, red diamonds), and solvent-separated ion pairs (SSIP, violet pentagons) with the $Cl^-$ anion. The error bars were computed using bootstrapping.

### Supplementary Note 7: Effect of the local solvation shell structure on the electric field gradient variance

The changes in the EFG variance $\langle \mathbf{V}^2 \rangle$, which determines the strength of the quadrupolar relaxation, are typically associated with the modification of the hydration shell structure and its symmetry [23–25]. For example, Versmold [23] demonstrated that the EFG at the position of a solute embedded into a dipolar liquid profoundly depends on the symmetry of the solvation shell and can be significantly reduced due to mutual cancellations for highly symmetric arrangements (e.g., in the case of tetrahedral or octahedral solvation shell structures). Furthermore, as the EFG is a rather short-range quantity that is proportional to $r^{-3}$ and $r^{-4}$ at the distance $r$ away from a point charge and dipole, respectively, the dominant contribution to $\langle \mathbf{V}^2 \rangle$ is due to molecules located in the immediate neighborhood of the solute [8].

To elucidate the effect of the solvation shell structure of $^{23}Na^+$ on the EFG variance at its position, in Fig. 7 we consider the EFG variance $\langle \mathbf{V}^2(N_{\mathrm{w}}) \rangle$ averaged over configurations with a different number of water molecules $N_{\mathrm{w}}$ in its first solvation shell for different concentrations and temperatures. The sodium ion has a quite small ionic radius of 1.02 Å that results in strong interactions with neighboring water molecules and the development of multiple coordination shell structures [21, 26]. As seen in Figs. 7**a** and 7**b** and as discussed in Refs. [21, 26], the latter are dominated by the square pyramidal and tri-

angular bipyramidal complexes coordinated by $N_\mathrm{w} = 5$ water molecules, as well as by the 6-coordinated square bipyramidal state. The much less likely tetrahedral structures with $N_\mathrm{w} = 4$ are also present. In agreement with recent machine learning based simulations parameterized on the strongly constrained and appropriately normed functional [21, 22], we find that the hydration shell state with $N_\mathrm{w} = 6$ is the most likely at low salt concentrations, yet is becoming somewhat less probable with increasing $c$ at the expense of tetrahedral structures with $N_\mathrm{w} = 4$ as well as complexes with $N_\mathrm{w} = 5$ (Fig. 7**a**). The latter rearrangements are due to ions that are more likely to penetrate into the first two sodium's hydration shells with increasing $c$ (see Figs. 3). Qualitatively similar trends are seen with decreasing $T$ (Fig. 7**b**). Consistently at different concentrations and temperatures, we find that the octahedral hydration complexes formed by 6 water molecules in the first solvation shell feature a reduced value of the EFG variance at the $Na^+$ position relative to the ensemble average $\langle \mathbf{V}^2 \rangle$ (Figs. 7**c** and 7**d**). While consistent with the Versmold's symmetry argument [23], the magnitude of the reduction at hand does not exceed 10% in comparison to $\langle \mathbf{V}^2 \rangle$. Furthermore, the EFG variance computed for less coordinated polyhedra with $N_\mathrm{w} = 4$ and 5 are about 10% larger than $\langle \mathbf{V}^2 \rangle$. This highlights that the changes of the QCC in different $Na^+$ hydration complexes are rather small within the considered range of salt concentrations and temperatures. Nonetheless, the trend of the variance reduction in sodium's octahedral hydration complexes can be understood on the basis of EFG cancellations in highly symmetric environments [23].

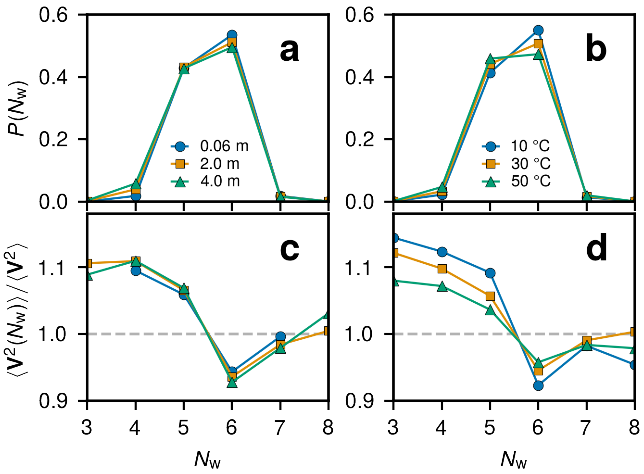


**Supplementary Figure 7**. **EFG variance in relation to the changes in the solvation shell structure of $Na^+$ ions.** Probability $P(N_\mathrm{w})$ of finding $N_\mathrm{w}$ water molecules in the first solvation shell of $Na^+$ ions for (**a**) increasing the salt concentration at $T = 25$ °C and for (**b**) decreasing temperature at $c = 1$ m. EFG variance corresponding to the states that contain $N_\mathrm{w}$ water molecules in the first solvation shell, $\langle \mathbf{V}^2(N_\mathrm{w}) \rangle$, relative to $\langle \mathbf{V}^2 \rangle$, for (**c**) increasing $c$ and (**d**) decreasing $T$.

### Supplementary Note 8: Solvation shell resolved electric field gradient relaxation

In this section, we discuss the origin of the EFG fluctuations in terms of the microscopic environment of the $Na^+$ ion. In Supplementary Fig. 8, we show the EFG ACFs at $T = 25$ °C and multiple concentrations, as computed from all point charges in the system using Ewald summation, from water molecules and ions residing in the first solvation shell only, and from water molecules and ions within the first two solvation shells. We find that the EFG ACF due to the first solvation shell decays much more slowly than the full EFG ACF, in particular with increasing salt concentration. In contrast, the EFG ACF due to point charges in the first two solvation shells provides a good description of the relaxation with the corresponding ACF capturing well both the short- and long-time dynamics (compare blue and black lines in Fig. 8). This indicates that the processes in the first two solvation shells are mainly responsible for the quadrupolar relaxation dynamics.

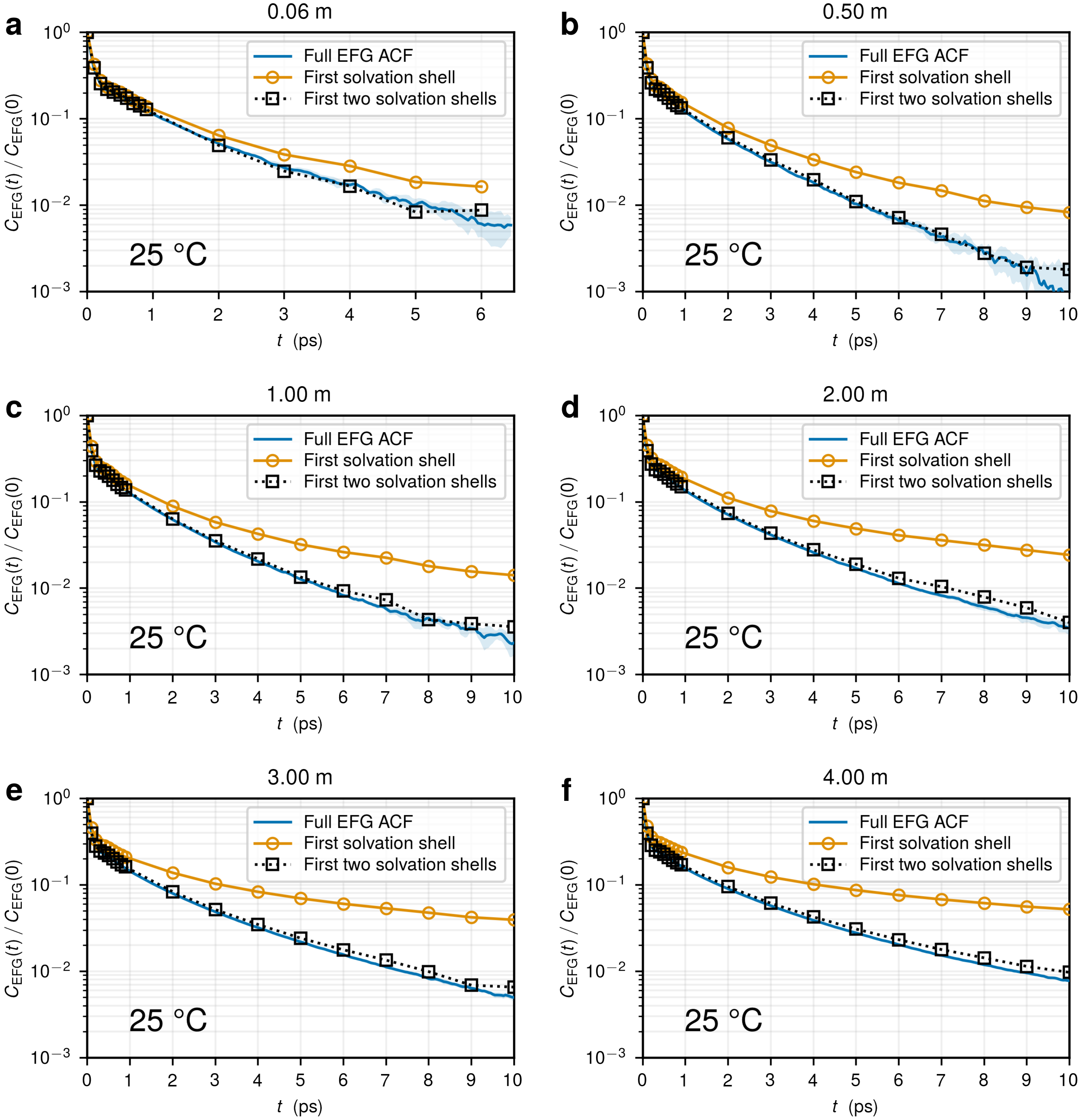


**Supplementary Figure 8**. EFG ACFs at the position of $Na^+$ ions in aqueous NaCl solutions at different salt concentrations (indicated in the plot titles) and $T$ = 25 °C, as computed from all point charges in the system (blue solid lines), from water molecules and ions in the first (solid yellow lines with open circles) and the first and second (black dashed lines with open squares) solvation shells. The shaded regions indicate the standard error from multiple independent simulation runs.

### Supplementary Note 9: Fitting of electric field gradient relaxation functions

In this section, we present additional results concerning the relaxation dynamics of the EFG at the position of $Na^+$ ions. In particular, in Fig. 9 we highlight the EFG ACFs for different temperatures and concentrations that supplement the results shown in the main text. Systematically for both the dilute and concentrated regimes, we find that the EFG ACFs have a characteristic form that consists of two steps: (*i*) fast initial decorrelation at short times ($t \lesssim 0.2$ ps); (*ii*) a slower decay on the ps time scale. While the initial fast decay barely changes with $c$ and $T$, as seen in Fig. 10 that shows the EFG ACFs for $t < 1.5$ ps, the main change in the EFG relaxation dynamics is reflected on the second decay of the ACFs that profoundly slows down with decreasing $T$ and with increasing salt concentration $c$. The latter is particularly evident at high salt concentrations (see Fig. 9**d-f**).

The effective EFG correlation time $\tau_c$ (11) is obtained directly from MD data by integrating the normalized ACFs. The running integrals of $C_{\mathrm{EFG}}(t)/C_{\mathrm{EFG}}(0)$ are shown in Fig. 16. Due to a pronounced slow-down of EFG fluctuations with increasing $c$ and decreasing $T$, the integration up to ∼50 ps may be necessary (e.g., at $T = 10$ °C and $c = 4$ m) to obtain well-converged values of $\tau_c$. This again highlights the importance of long-time sampling in the description of the NMR relaxation rates in concentrated electrolyte solutions.

To assess the EFG relaxation behavior, we attempted to fit the normalized EFG ACFs, $C_{\mathrm{EFG}}(t)/C_{\mathrm{EFG}}(0)$, using different functional relations. Given a two-step shape of the relaxation decay, we first modelled the EFG ACFs using a sum of two exponentials:

$$f_1(t) = (1-\alpha)e^{-t/\tau_{\mathrm{f}}} + \alpha e^{-t/\tau_1}, \qquad (21)$$

where $\tau_{\mathrm{f}}$ is the time scale of the initial, fast process that occurs at $t \lesssim 0.2$ ps, $\tau_1$ is the time scale of the second, slow process, and $\alpha$ is its relative weight. The resulting fits using Eq. (21) are shown with dotted lines in Fig. 9 for different salt concentrations and temperatures (the average $R^2$ score is 0.995 over the ensemble of fitted curves). Furthermore, in Fig. 10 the same fits and EFG ACFs are plotted for $t < 1.5$ ps. Evidently, for both dilute salt concentrations (Fig. 9**a**) and in the concentrated regime (Fig. 9**b-f**) the two exponential fit (21) is only valid for $t \lesssim 2{-}3$ ps, whereas the consecutive decay at longer times is slower. The latter highlights the non-exponential character of the relaxation and points to a collective pathway behind the EFG relaxation. Quantitatively, by performing a generic three-parameter fit in Eq. (21), we find that $\tau_{\mathrm{f}}$ features small changes with respect to $c$ and $T$, varying between 58 and 65 fs. At $T = 25$ °C, $\tau_{\mathrm{f}}$ adopts a value of around 62 fs at the lowest concentration considered and decreases to ∼60 fs at $c = 4$ m. The parameter $\alpha$ also features rather small changes and has values in range between 0.26 and 0.3, growing with increasing $c$ and decreasing $T$. The main changes with $c$ and $T$ are reflected on the time scale of the second decay mode $\tau_1$ that considerably rises with with increasing $c$ and decreasing $T$. At $T = 25$ °C, $\tau_1$ changes from 1.16 ps at $c = 0.06$ m to 1.62 ps at $c = 4$ m, an overall increase by a factor of ∼1.4. At the lowest $c$ here, $\tau_1$ changes from 0.81 ps to 1.55 ps by decreasing $T$ from 50 to 10 °C.

To improve the representation of EFG ACFs at long times, we have included an additional second slow process with a time scale $\tau_2$ and weight $\alpha_2$ in the fitting procedure:

$$f_2(t) = (1-\alpha_1-\alpha_2)e^{-t/\tau_{\mathrm{f}}} + \alpha_1 e^{-t/\tau_1} + \alpha_2 e^{-t/\tau_2}. \qquad (22)$$

To reduce the number of independent fitting parameters in Eq. (22), we reused the values of $\tau_{\mathrm{f}}$ obtained in the fitting with Eq. (21). As seen in Fig. 11, the three exponential fit (21) provides a quite good representation of the relaxation dynamics of EFG fluctuations for most of the decay (more than two orders of magnitude relative to the initial value of the ACF, roughly up to ∼10 ps). The associated average $R^2$ score is 0.999 over the ensemble of fitted curves. Yet, the presence of even slower relaxation modes is evident in the concentrated solutions at very long times (Fig. 11**d-f**). For the considered $c$ and $T$, the relative weight $\alpha_1$ attains values 0.15-0.21, whereas $\alpha_2$ around 0.1–0.14. Both time constants $\tau_1$ and $\tau_2$ grow with increasing $c$ and decreasing $T$. For instance, at $T = 25$ °C, $\tau_1$ increases from 0.65 to 1.1 ps, whereas $\tau_2$ from 2.21 to 3.95 ps. Since the fit (22) provides a good description of the bulk part of the decay, the effective EFG correlation times obtained by integrating the model expression (22), $\tau_{\mathrm{c,\,three\,exp.}} = (1-\alpha_1-\alpha_2)\tau_{\mathrm{f}} + \alpha_1\tau_1 + \alpha_2\tau_2$, is in good agreement with $\tau_c$ obtained by directly integrating the EFG ACFs from MD.

We find that the EFG ACFs can be equally well represented by a simpler fit that is composed of a single exponential to model the fast initial decay and a stretched exponential to model to consecutive, slower part of the relaxation:

$$f_3(t) = (1-\alpha_{\mathrm{s}})e^{-t/\tau_{\mathrm{f}}} + \alpha_{\mathrm{s}} e^{-[t/\tau_{\mathrm{s}}]^{\beta}}, \qquad (23)$$

where $\alpha_{\mathrm{s}}$ is the relative weight of the stretched exponential function, $\tau_{\mathrm{s}}$ is its time scale, and $\beta$ is the stretching exponent. While the expression (23) generally consists of four independent fitting parameters, certain approximation can be applied: (*i*) we reuse the values of $\tau_{\mathrm{f}}$ obtained in the fitting of Eq. (21); (*ii*) we obtain the value of $\beta = 0.67 \pm 0.05$ by fitting the tail of the EFG ACF at the lowest concentration considered, $c = 0.06$ m, and fix it in the following optimizations. This reduces the fit (23) to just two independent parameters, $\alpha_{\mathrm{s}}$ and $\tau_{\mathrm{s}}$. The resulting fits shown with dotted lines are in good agreement with the measured EFG ACFs, as seen in Fig. 12 for multiple temperatures and concentrations. The average $R^2$ score is 0.998. Similarly to the three-exponential fit (22), the model employing the stretched exponential function (23) decays faster at long times in comparison

to the EFG ACFs obtained in MD. This might indicate that the long-time EFG relaxation decay is slower than both exponential and stretched exponential relations. In fact, in the main text we show that the long-time tail of the EFG ACFs is consistent with a power-law $\sim t^{-5/2}$ that was predicted within a mode coupling theory [19].

In Fig. 13, we show the concentration and temperature dependence of the optimized values of $\tau_{\rm s}$ and $\alpha_{\rm s}$ (Fig. 13**a** and 13**b**, respectively) of the fit in Eq. (23). The parameter $\tau_{\rm s}$ mimics the behavior of $\tau_{\rm c}$, yet adopts values that are about twice as high. $\alpha_{\rm s}$, somewhat decreasing with increasing $c$ and $T$, features values in range between 0.35 and 0.39. By integrating the expression (23), we find the effective, predicted EFG correlation time

$$\tau_{\rm c,stretched} = (1-\alpha_{\rm s})\tau_{\rm s} + \beta^{-1}\Gamma(\beta^{-1})\alpha_{\rm s}\tau_{\rm s}, \tag{24}$$

with $\Gamma(x)$ denoting the gamma function. The values of $\tau_{\rm c,stretched}$ are in excellent agreement with the $\tau_{\rm c}$ results obtained by directly integrating the ACFs from MD. Finally, we find that the stretched exponential in Eq. (23) provides a dominant contribution to $\tau_{\rm c}$. The latter is highlighted in Fig. 13**d** where we show $\beta^{-1}\Gamma(\beta^{-1})\alpha_{\rm s}\tau_{\rm s}$ scaled with $\tau_{\rm c}$. It is evident that the second, slow part of the EFG ACF decay constitutes more than 85% of $\tau_{\rm c}$, and its contribution increases with increasing $c$ and decreasing $T$.

The stretched exponential decay $\sim e^{-[t/\tau_{\rm s}]^{\beta}}$ can be interpreted as a continuous superposition of exponential modes with a different rate constant $k$ [27]:

$$e^{-[t/\tau_{\rm s}]^{\beta}} = \int_0^{+\infty} {\rm d}k\, H(k) e^{-kt}, \tag{25}$$

where $H(k)$ is the distribution function that quantities the relative weight of each $k$-mode, satisfying $\int_0^{+\infty} {\rm d}k\, H(k) = 1$. Formally, $H_\beta(k)$ can be obtained as the inverse Laplace transform of $e^{-[t/\tau_{\rm s}]^{\beta}}$. Analytical, closed-form expressions were derived for certain rational $\beta$, e.g. 1/3 and 2/3 [27]. Moreover, there exists a convergent series that can be used to determine $H_\beta(k)$ in the general case [27]. Nevertheless, the latter series features an oscillating behavior that complicates its use in practical numerical calculations [27]. Thus, to understand the behavior of $H(k)$ in our case of the EFG ACFs (23), we have employed the approximate numerical expressions of Berberan et al. [27]:

$$\begin{aligned} H_\beta(k) = \tau_{\rm s}\, &\frac{B}{(k\tau_{\rm s})^{(1-\beta/2)/(1-\beta)}} \times \\ &\times \exp\left[-\frac{(1-\beta)\beta^{\beta/(1-\beta)}}{(k\tau_{\rm s})^{\beta/(1-\beta)}} f(k)\right], \end{aligned} \tag{26}$$

where $f(k) = 1/(1+C(k\tau_{\rm s})^{\delta})$ with $\delta = \beta(0.5-\beta)/(1-\beta)$ for $\beta \leq 0.5$, and $f(k) = 1 + C(k\tau_{\rm s})^{\delta}$ with $\delta = \beta(\beta - 0.5)/(1-\beta)$ for $\beta > 0.5$. For $\beta = 0.67$, the numerical constants $B$ and $C$ have values $B = 0.341595$ and $C = 0.1833925$. The resulting $H(k)$ for the stretched exponential contribution in the fit (23) are shown in Fig. 14. Both for increasing $c$ at a fixed $T$ (Fig. 14**a**) and for decreasing $T$ at a fixed $c$ (Fig. 14**c**), $H(k)$ features a rather broad distribution with a single peak, whose maximum position shifts towards larger $k$. The inverse of the peak position of $H(k)$, $k_{\rm max}^{-1}$, (Fig. 14**b** and 14**d**) generally follows the behavior of $\tau_{\rm c}$ and adopts values between 1 and 5 ps, typically 2-3 times larger than the actual value of the effective EFG correlation time $\tau_{\rm c}$.

In Fig. 15, we additionally highlight the long-time behavior of the EFG ACFs by multiplying it by $t^{5/2}$ and $t^{3/2}$. As seen in Fig. 15**a**, $t^{5/2}\, C_{\rm EFG}(t)$ saturates at long times, suggesting an algebraic decay $\sim t^{-5/2}$ that is consistent with the mode coupling theory of Bosse et al. [19].

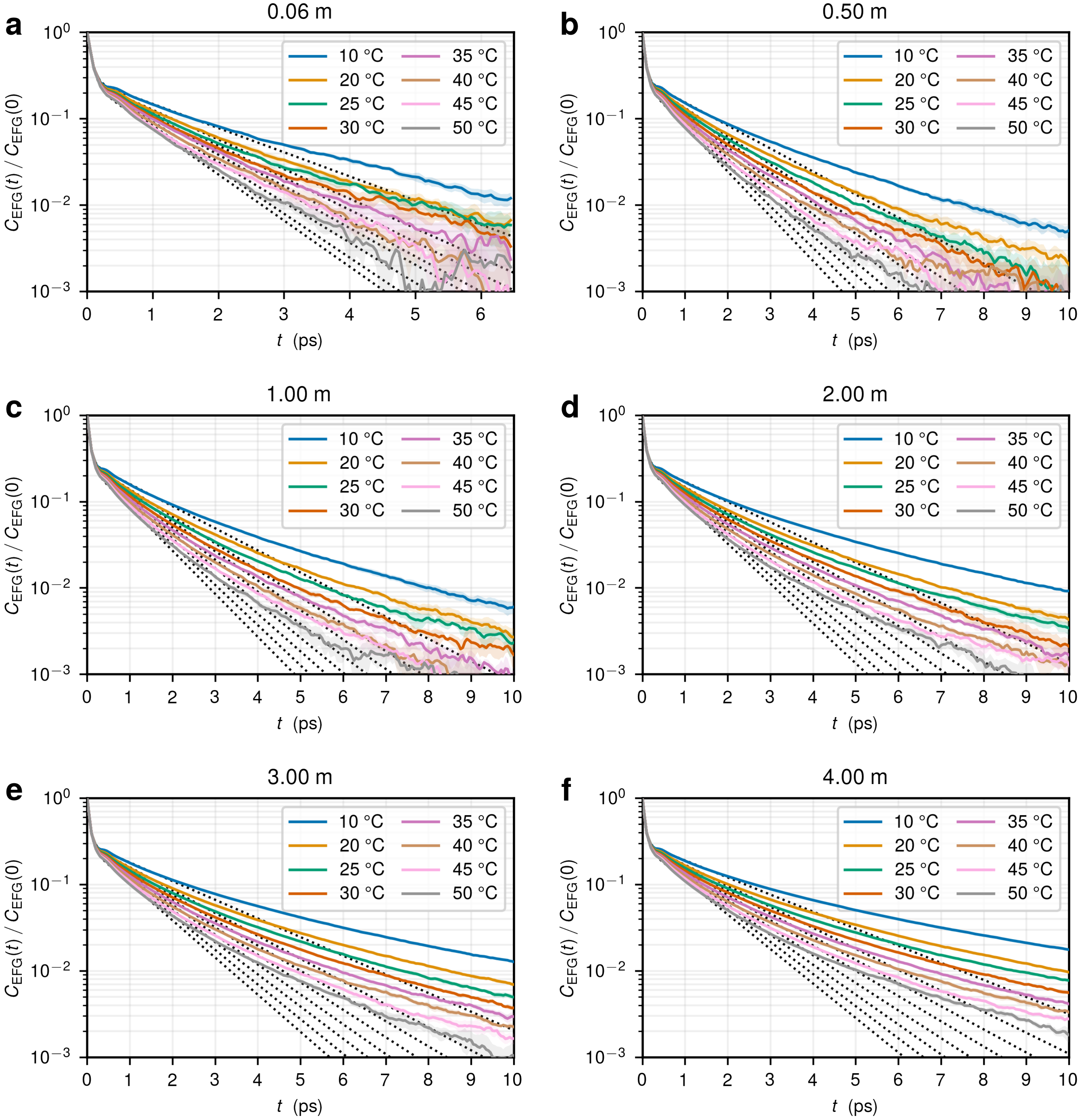


**Supplementary Figure 9**. EFG ACFs at the position of $Na^+$ ions in aqueous NaCl solutions for different temperatures (indicated in the legend) and salt concentrations (indicated in the plot titles). The dotted lines correspond to fits with a sum of two exponentials in Eq. (21). The shaded regions indicate the standard error from multiple independent simulation runs.

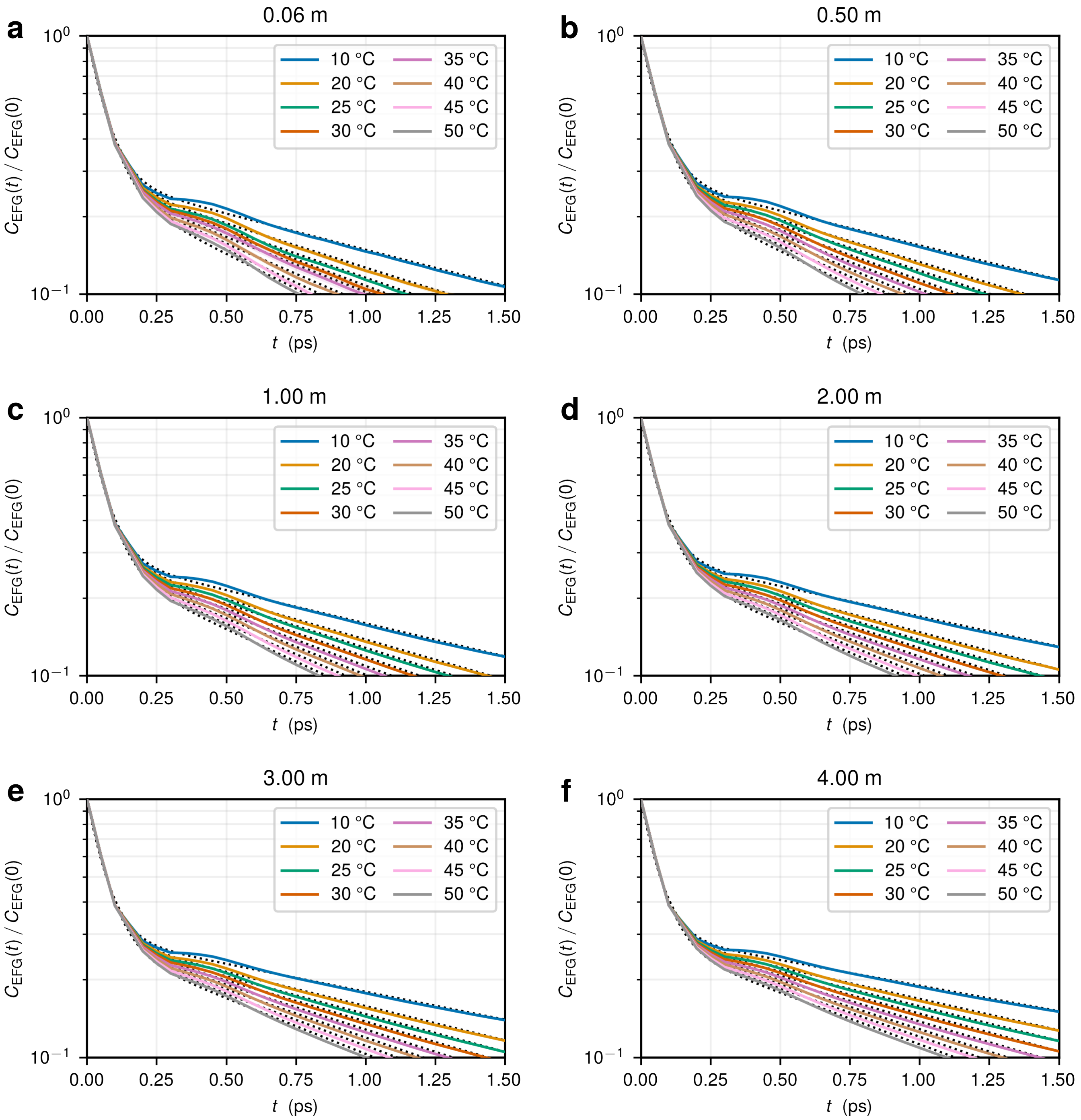


**Supplementary Figure 10**. EFG ACFs at the position of $Na^+$ ions in aqueous NaCl solutions for different temperatures (indicated in the legend) and salt concentrations (indicated in the plot titles) shown for $t < 1.5$ ps. The dotted lines correspond to fits with a sum of two exponentials in Eq. (21).

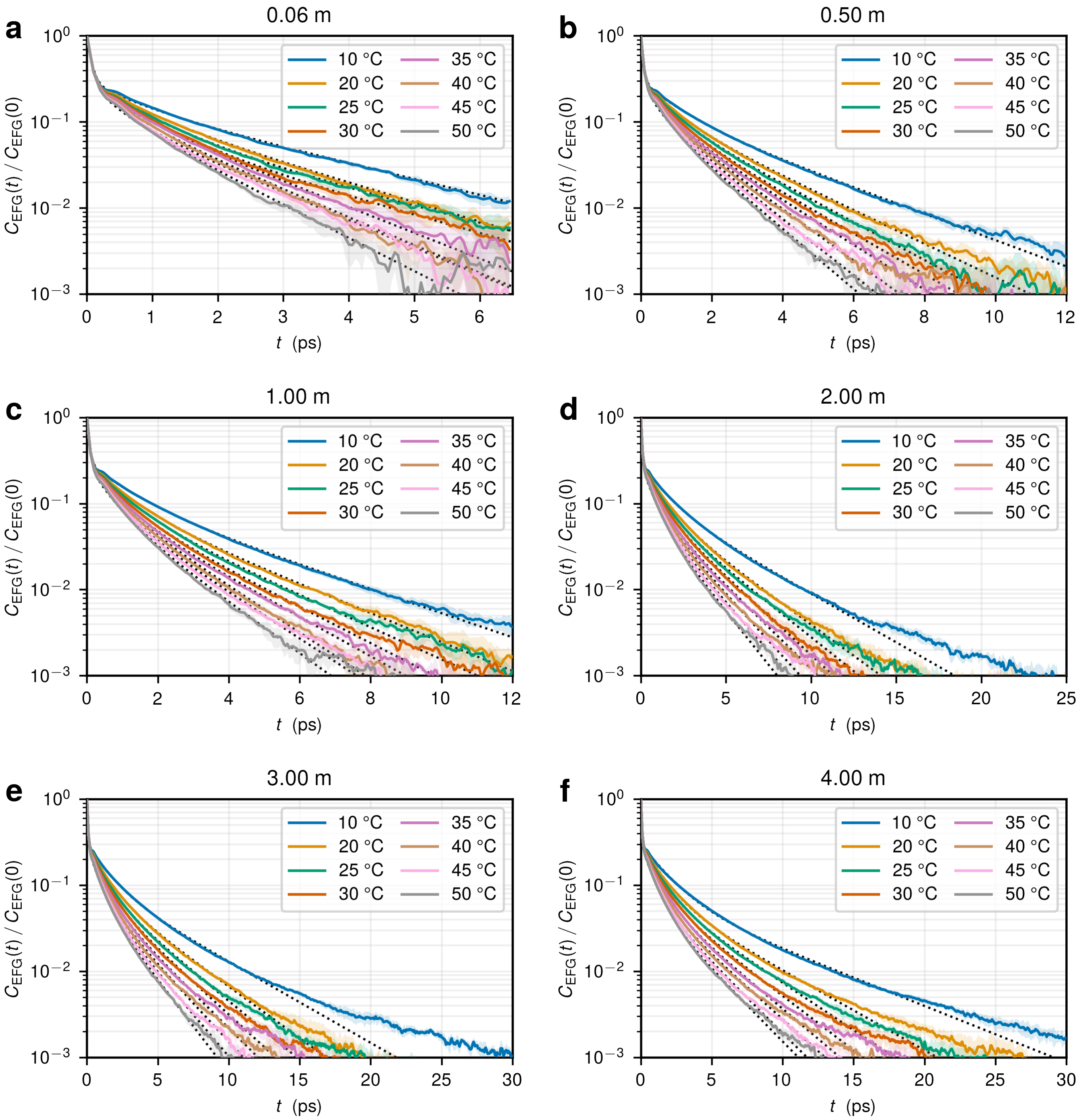


**Supplementary Figure 11**. EFG ACFs at the position of $Na^+$ ions in aqueous NaCl solutions for different temperatures (indicated in the legend) and salt concentrations (indicated in the plot titles). The dotted lines correspond to fits with a sum of three exponentials in Eq. (22). The shaded regions indicate the standard error from multiple independent simulation runs.

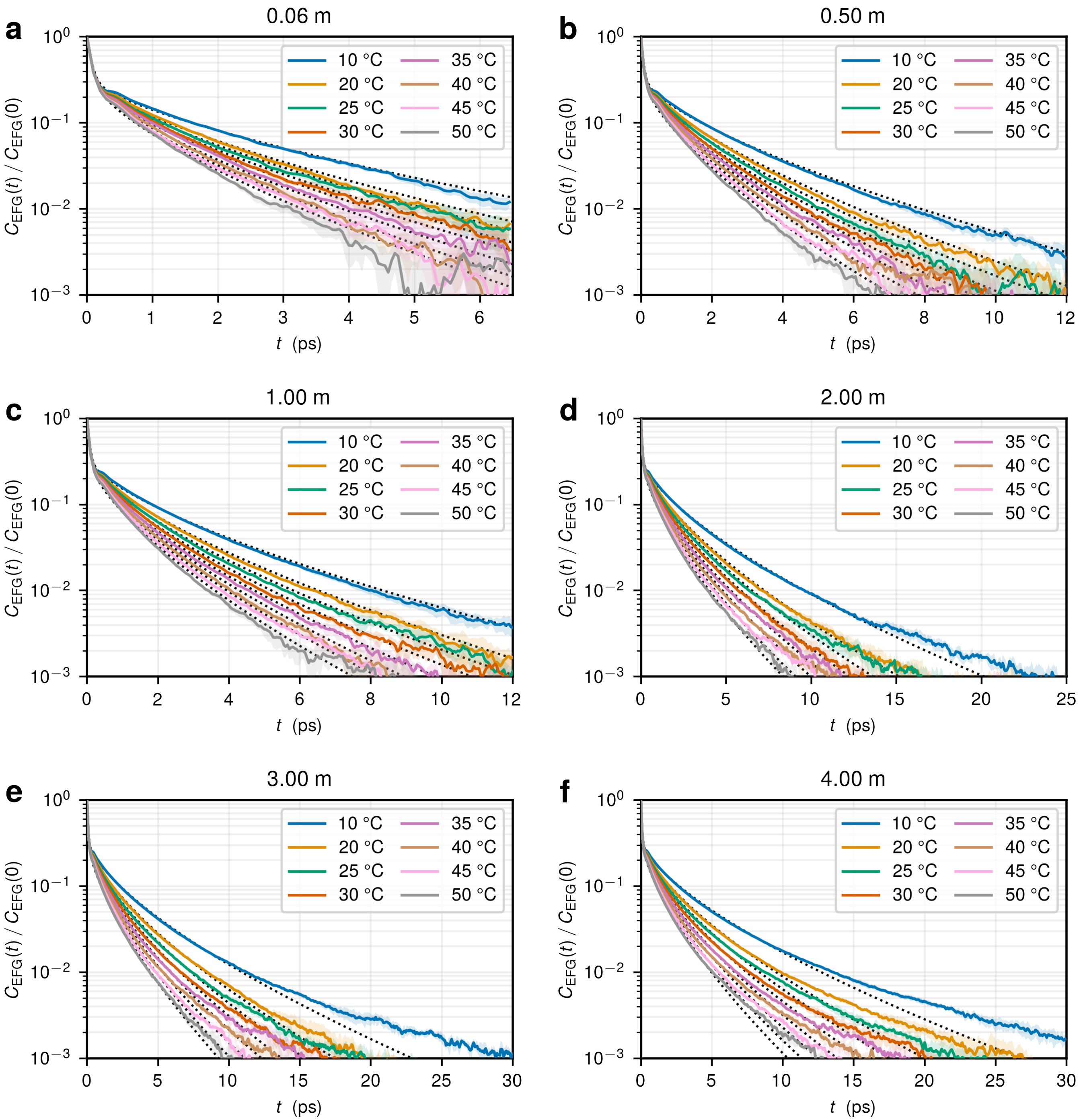


**Supplementary Figure 12**. EFG ACFs at the position of $Na^+$ ions in aqueous NaCl solutions for different temperatures (indicated in the legend) and salt concentrations (indicated in the plot titles). The dotted lines correspond to fits with a sum of an exponential and a stretched exponential in Eq. (23). The shaded regions indicate the standard error from multiple independent simulation runs.

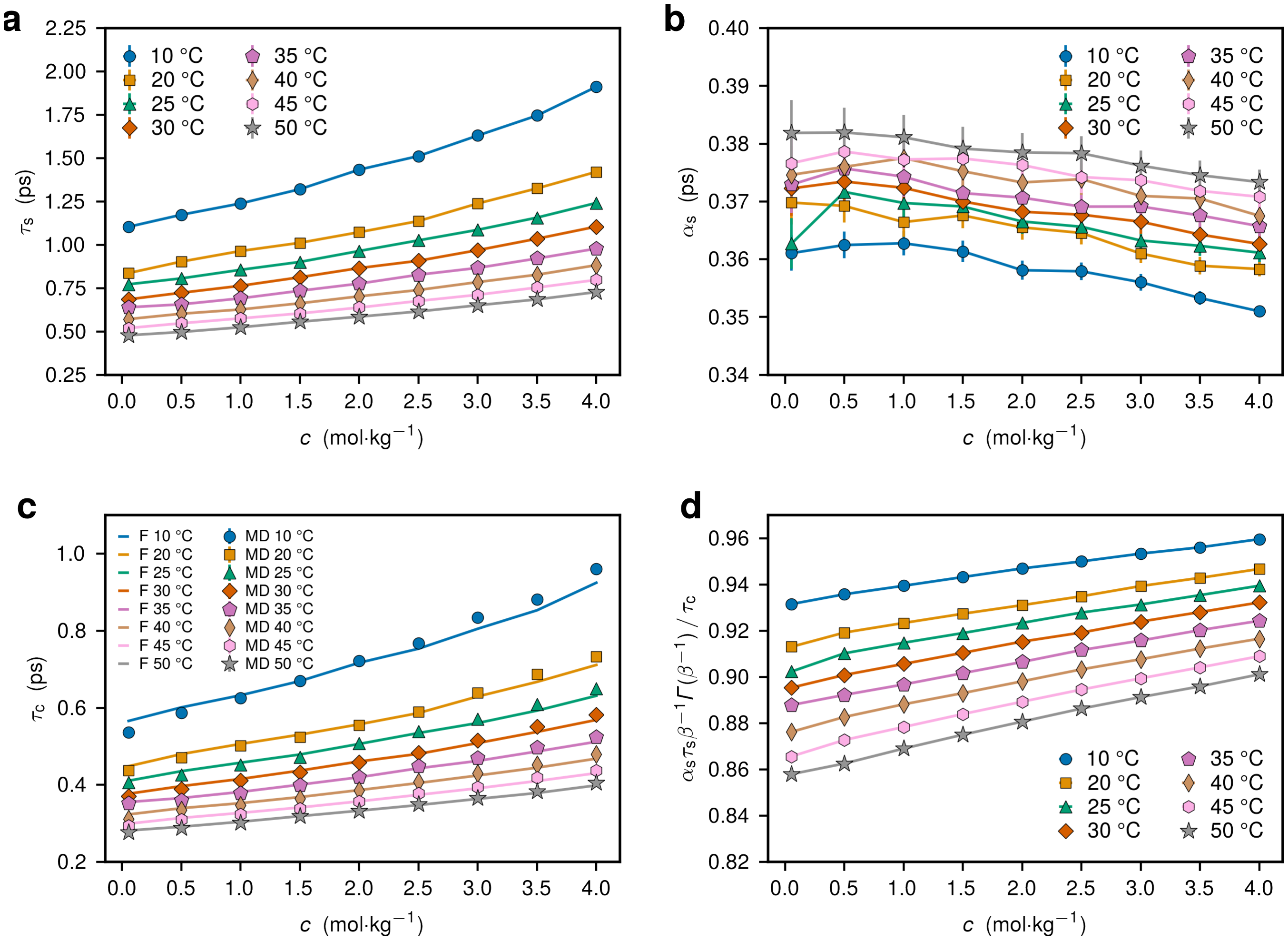


**Supplementary Figure 13**. Parameters of the fit in Eq. (23). **a** Time scale of the stretched exponential $\tau_{\mathrm{s}}$ as a function of the salt concentration $c$ for different temperatures $T$. **b** Weight of the stretched exponential contribution $\alpha_{\mathrm{s}}$ in the fit (23). **c** Effective EFG correlation time $\tau_{\mathrm{c}}$ as obtained directly in MD (filled symbols) and by integrating the fitting expression (23) (solid lines). **d** Contribution of the stretched exponential $\alpha_{\mathrm{s}}\tau_{\mathrm{s}}\beta^{-1}\Gamma(\beta^{-1})$ to $\tau_{\mathrm{c}}$. The standard error from multiple independent simulation runs is either explicitly shown or does not exceed the symbol size.

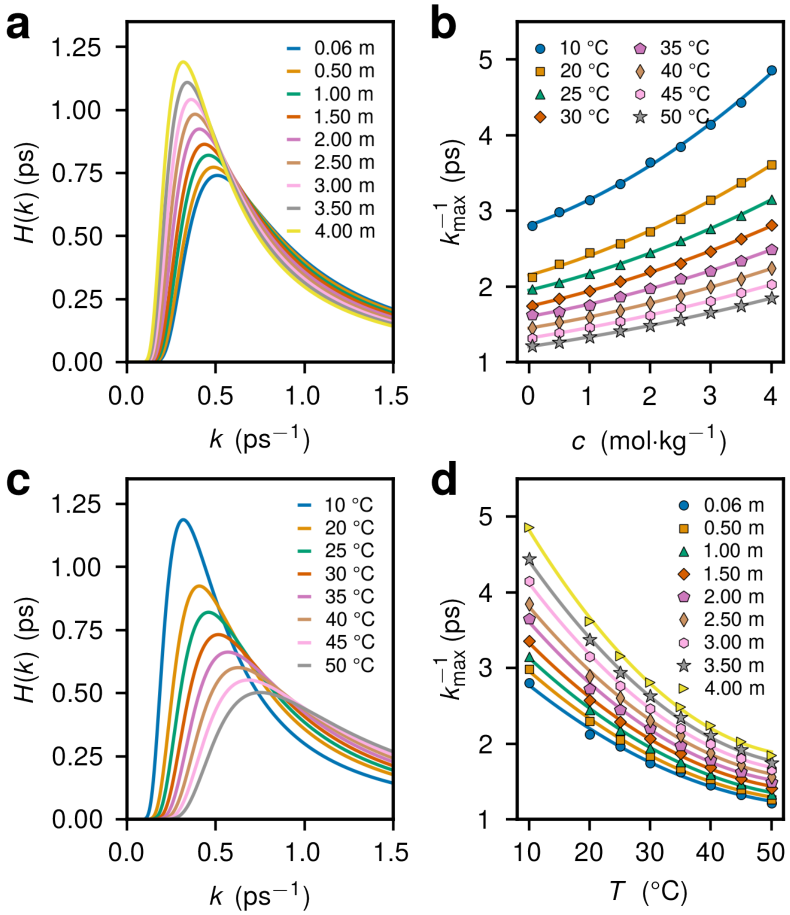


**Supplementary Figure 14**. Decomposition of the stretched exponential in terms of single exponentials with rate constants $k$, as given in Eq. (25). Distribution of rate constants $H(k)$ as a function of $k$ for different salt concentrations at $T = 25$ °C (**a**) and temperatures at $c = 1$ m (**c**). Inverse of the position of the peak of $H(k)$, $k_{\rm max}^{-1}$, as a function of $c$ (**b**) and $T$ (**d**).

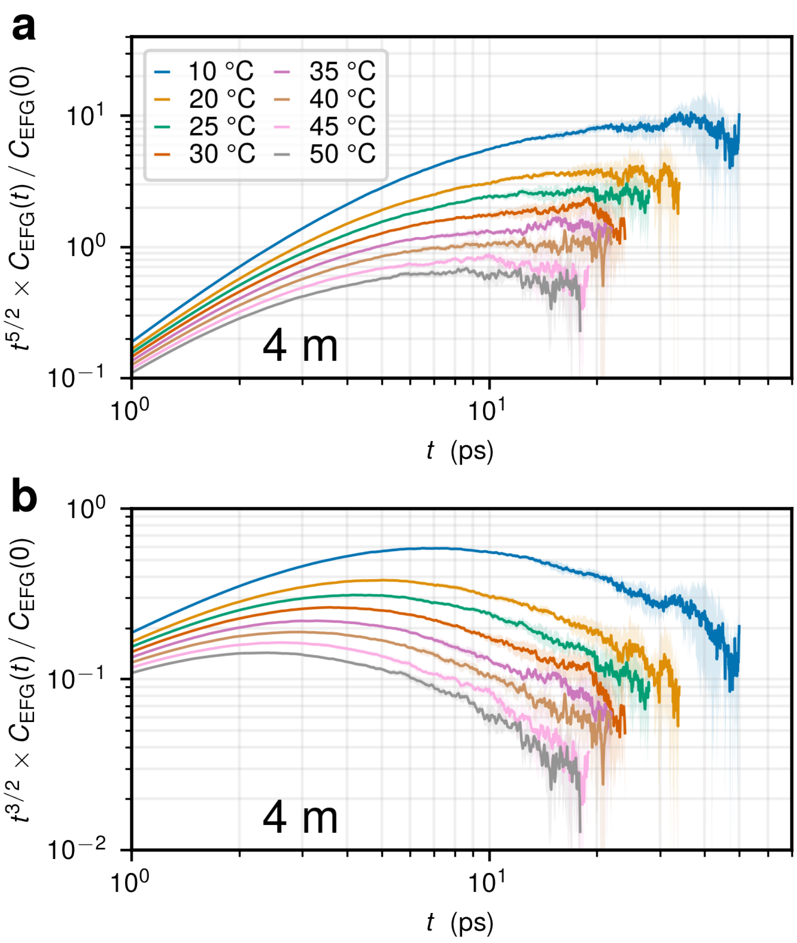


**Supplementary Figure 15**. Normalized EFG ACFs multiplied by (**a**) $t^{5/2}$ and (**b**) $t^{3/2}$ for $^{23}Na^{+}$ ions in aqueous NaCl solutions at $c = 4$ m and different temperatures (indicated in the legend). The shaded regions indicate the standard error from multiple independent simulation runs.

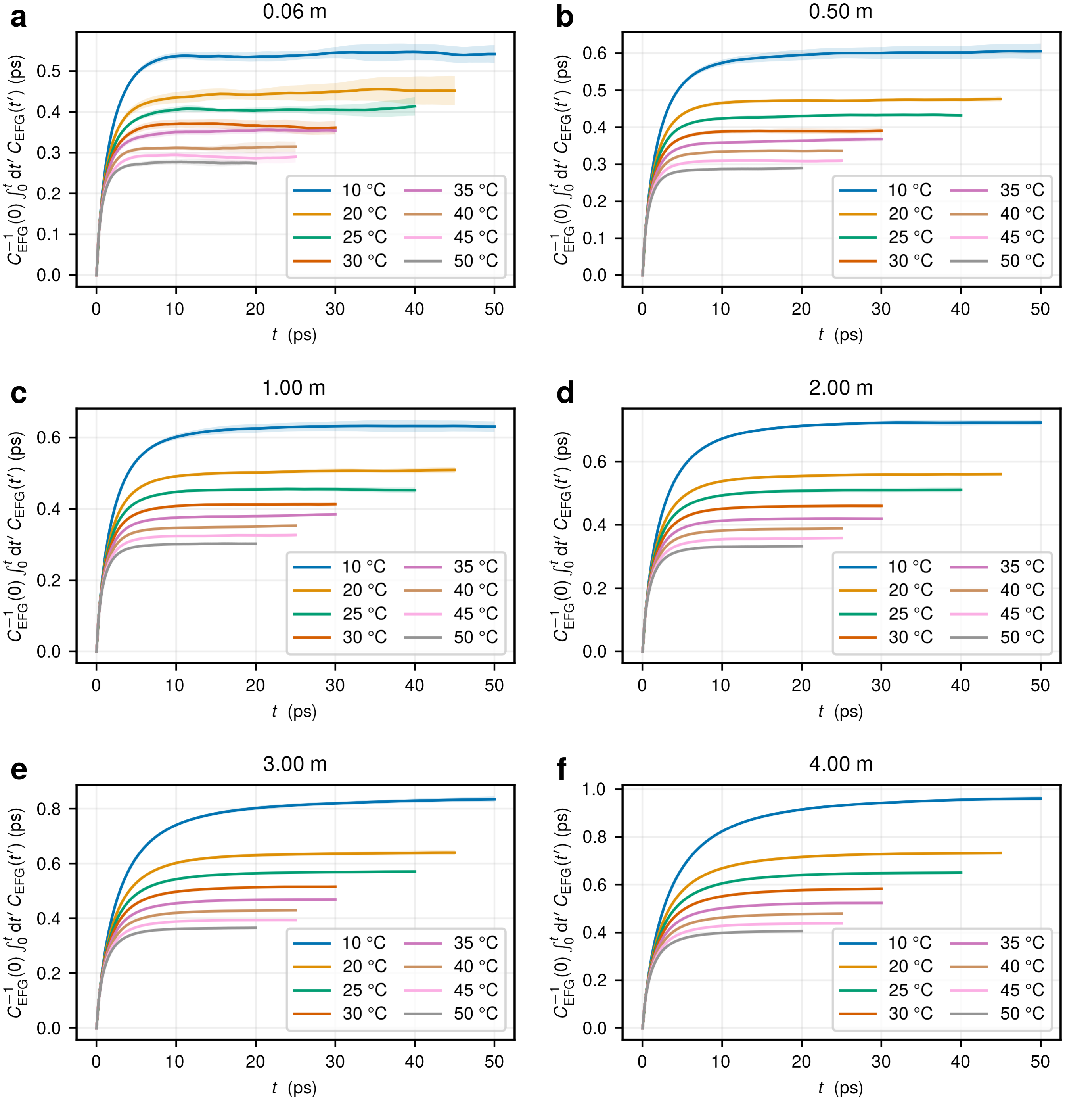


**Supplementary Figure 16**. Time-dependent integrals the normalized EFG ACFs at the position of $Na^+$ ions in aqueous NaCl solutions for different temperatures (indicated in the legend) and salt concentrations (indicated in the plot titles). The shaded regions indicate the standard error over different simulation runs.

### Supplementary Note 10: Quadrupolar coupling constant

In Fig. 17, we show the concentration and temperature dependence of the QCC $C_Q$ that is related to the EFG variance $\langle \mathbf{V}^2 \rangle$ through Eq. (14) as follows:

$$C_Q = \sqrt{\frac{2\langle \mathbf{V}^2 \rangle}{3}} \left(\frac{eQ}{\hbar}\right) \tag{27}$$

$C_Q$ decreases with increasing $c$ and decreasing $T$ in range from around $19 \times 10^6$ to $20.6 \times 10^6$ rad·s$^{-1}$. At $T = 25$ °C and low $c$, $C_Q = 19.87 \times 10^6$ rad·s$^{-1}$.

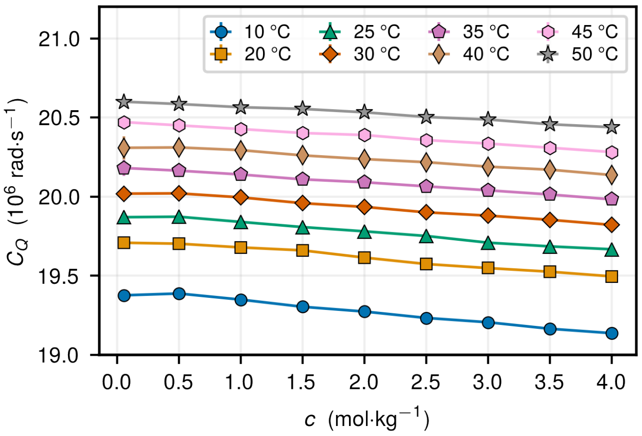


**Supplementary Figure 17**. QCC given by Eq. (14) for $^{23}Na^+$ ions in aqueous NaCl solutions as a function of the salt concentration $c$ for different temperatures indicated in the legend.

### Supplementary Note 11: Water dipole reorientation

We quantify the mean time scale $\tau_{\text{dip}}$ of water dipole reorientation by means of the integral

$$\tau_{\text{dip}} = \int_0^\infty \mathrm{d}t \, \langle P_1[\mathbf{u}(t) \cdot \mathbf{u}(0)] \rangle, \tag{28}$$

where $P_1(x) = x$ is the first Legendre polynomial and $\mathbf{u}(t)$ is a unit vector at time $t$ that points in the direction of the dipole of a TIP4P/2005 water molecule (i.e., from the virtual M-site towards the oxygen atom). The resulting concentration and temperature dependence of $\tau_{\text{dip}}$ is shown in Fig. 18.

### Supplementary Note 12: Quadrupolar relaxation within a Stokes-Einstein-Debye model

In Fig. 19**a** and 19**b**, we extract the hydrodynamics radius $r_0$ of $Na^+$ ions in aqueous NaCl solutions by means

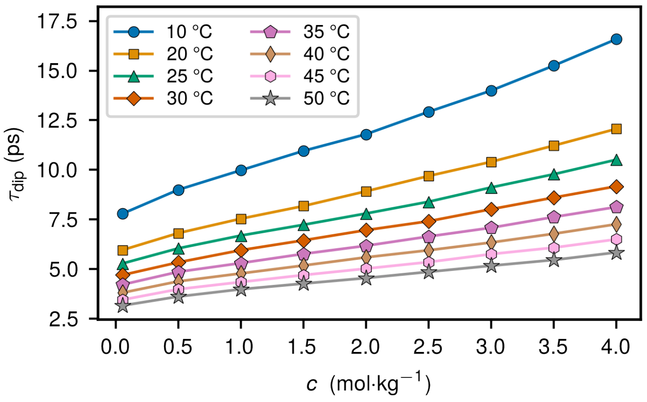


**Supplementary Figure 18**. The mean time $\tau_{\text{dip}}$ of water dipole reorientation, see Eq. (28), as a function of $c$ for different $T$ in aqueous NaCl solutions.

of the Stokes-Einstein relaxation $r_0 = k_B T / 6\pi\eta D$ using the data from experiments and simulations, respectively. The values of $r_0$ are generally between 1.5 and 2.0 Å for considered salt concentrations $c$ and temperatures $T$. The hydrodynamic radius tends to decrease with increasing $c$ and $T$. Complementary to Fig. 5 in the main text, in Fig. 19**c** we extract the Stokes-Einstein-Debye time scale $\tau_c^{\text{SED}} = 4\pi\eta r_0^3 / 3k_B T$ from the results in simulations. The resulting $\tau_c^{\text{SED}}$ are more than an order of magnitude larger than the respective EFG correlation time $\tau_c$. In agreement with the experimental results in Fig. 5, we find that $\tau_c$ in simulations satisfies a generalized Stokes-Einstein relation, $\tau_c \propto \eta / k_B T$ (Fig. 19**d**). The associated effective hydrodynamic radius that can be obtained using a fit $\tau_c = 4\pi\eta \left[r_0^{\text{eff}}\right]^3 / 3k_B T + \tau_0^{\text{eff}}$ is $r_0^{\text{eff}} = 0.68$ Å with $\tau_0^{\text{eff}} = 0.12$ ps.

### Supplementary Note 13: Quadrupolar relaxation of $^{23}Na^+$ in other electrolyte solutions

The EFG relaxation of $^{23}Na^+$ in NaBr, NaF, and NaCl is further discussed in Fig. 20 for different salt concentrations at $T = 25$ °C. The dynamics of EFG fluctuations for $^{23}Na^+$ in NaBr and NaF are quite similar to the case of NaCl discussed in detail in the main text, as we show with the normalized EFG ACF in NaBr for different salt concentrations in Fig. 20**a** and by comparing the EFG ACFs in NaCl, NaBr, and NaF at $c = 1$ m in Fig. 20**b**. Quite small differences in the slow part of the EFG ACFs (Fig. 20**b**) cause a discrepancy in the effective correlation time $\tau_c$ (Fig. 20**c**). Whereas the magnitude of the EFG variance at the ion position $\langle \mathbf{V}^2 \rangle$ features a small decrease with increasing $c$ (Fig. 20**d**), the lengthening of the effective correlation time $\tau_c$ (Fig. 20**c**) results in the increase of the quadrupolar relaxation rate (Fig. 20**e**) in all cases.

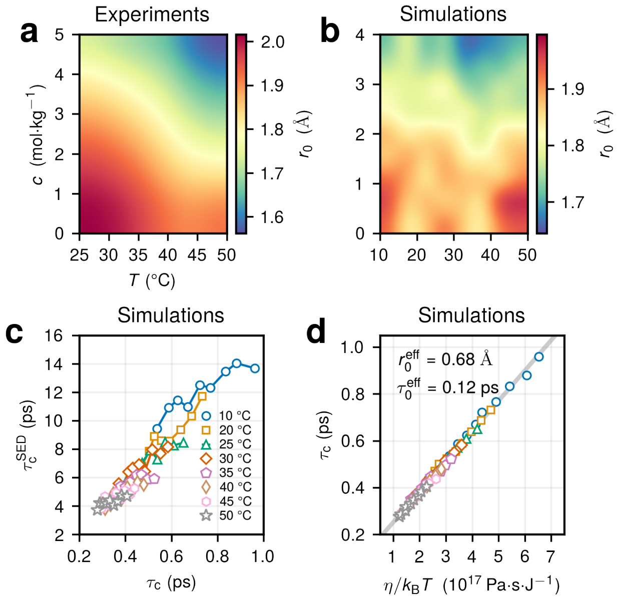


**Supplementary Figure 19**. **EFG correlation time within the Stokes-Einstein-Debye model.** Hydrodynamic radius of a $Na^+$ ion $r_0 = k_B T/6\pi\eta D$ in (**a**) experiments and (**b**) simulations for different salt concentrations $c$ and temperatures $T$. **c** Stokes-Einstein-Debye correlation time $\tau_c^{SED} = 4\pi\eta r_0^3/3k_B T$ plotted versus the EFG correlation time $\tau_c$ as extracted from the simulation data at different $c$ and $T$. **d** $\tau_c$ plotted versus $\eta/k_B T$ at different $c$ and $T$. The gray line shows the fit $\tau_c = 4\pi\eta\left[r_0^{eff}\right]^3/3k_B T + \tau_0^{eff}$ with the best fit parameters $r_0^{eff}$ and $\tau_0^{eff}$ indicated in the legend.

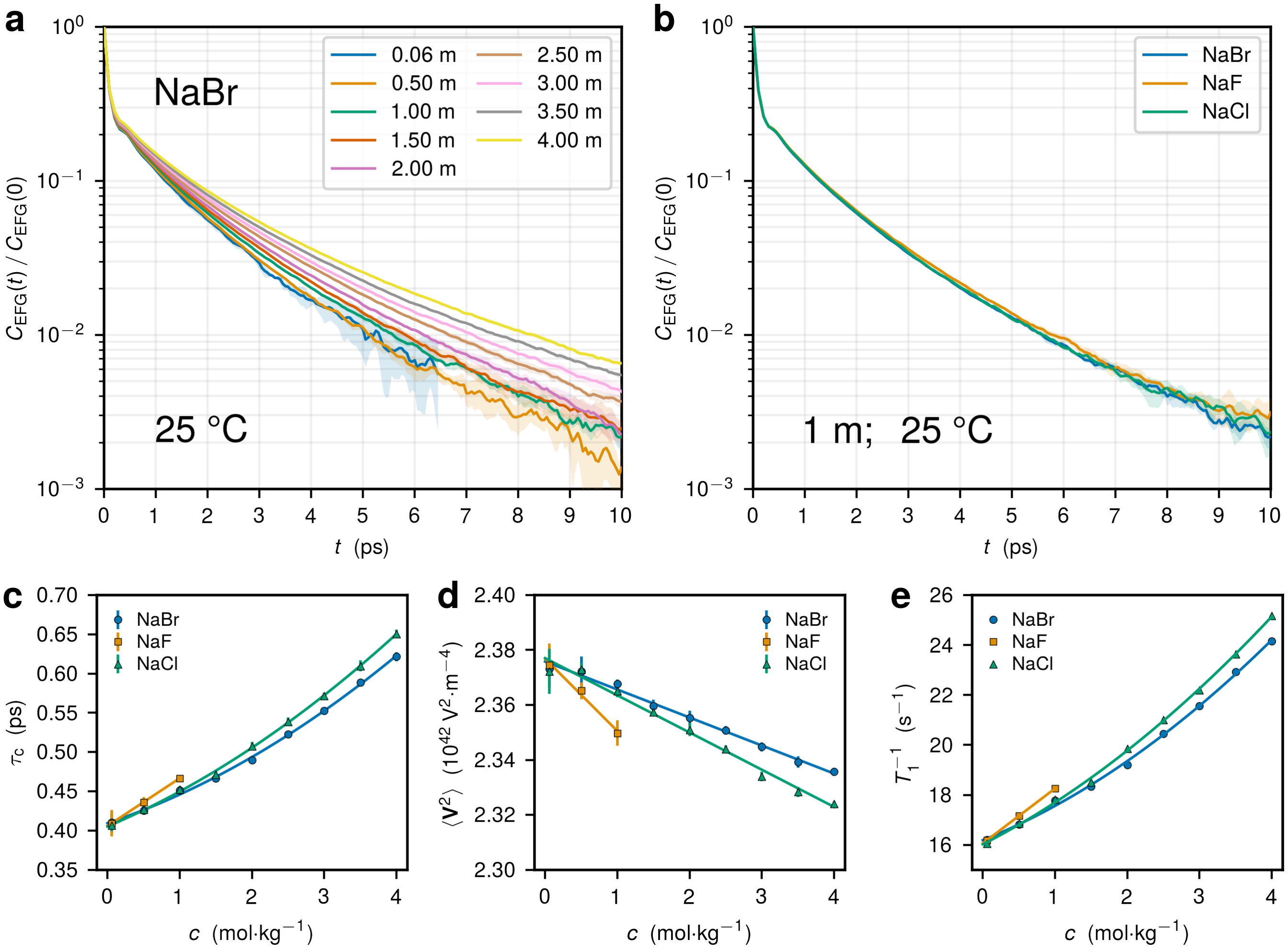


**Supplementary Figure 20**. EFG relaxation at the position of $Na^+$ ions in different electrolyte solutions at $T$ = 25 °C. **a** Normalized EFG ACFs at the position of $Na^+$ ions in aqueous NaBr solutions for different salt concentrations. **b** The EFG ACF for $Na^+$ in aqueous NaBr, NaF, and NaCl solutions for $c$ = 1 m and $T$ = 25 °C. In **a** and **b**, the shaded regions indicate the standard error from multiple independent simulation runs. The effective EFG correlation time $\tau_c$ (**c**) and the EFG variance (**d**) as a function of $c$ in NaBr, NaF, and NaCl solutions. **e** The resulting quadrupolar NMR relaxation rates for $^{23}Na^+$ in aqueous NaBr, NaF, and NaCl solutions as a function of the salt concentration $c$. In **c**, **d**, and **e**, the standard error from multiple independent simulation runs is either explicitly shown or does not exceed the symbol size.

## SUPPLEMENTARY REFERENCES


[1] Spiess, H. W. Rotation of molecules and nuclear spin relaxation. In *Dynamic NMR Spectroscopy*, 55–214 (Springer Berlin Heidelberg, Berlin, Heidelberg, 1978).

[2] Cowan, B. *Nuclear Magnetic Resonance and Relaxation* (Cambridge University Press, 1997).

[3] Badu, S., Truflandier, L. & Autschbach, J. Quadrupolar NMR spin relaxation calculated using ab initio molecular dynamics: Group 1 and group 17 ions in aqueous solution. *J. Chem. Theory Comput.* **9**, 4074–4086 (2013).

[4] Hubbard, P. S. Nonexponential nuclear magnetic relaxation by quadrupole interactions. *J. Chem. Phys.* **53**, 985–987 (1970).

[5] Zeron, I. M., Abascal, J. L. F. & Vega, C. A force field of $Li^+$, $Na^+$, $K^+$, $Mg^{2+}$, $Ca^{2+}$, $Cl^-$, and $SO_4^{2-}$ in aqueous solution based on the TIP4P/2005 water model and scaled charges for the ions. *J. Chem. Phys.* **151**, 134504 (2019).

[6] Blazquez, S., Conde, M. M., Abascal, J. L. F. & Vega, C. The Madrid-2019 force field for electrolytes in water using TIP4P/2005 and scaled charges: Extension to the ions $F^-$, $Br^-$, $I^-$, $Rb^+$, and $Cs^+$. *J. Chem. Phys.* **156**, 044505 (2022).

[7] Abragam, A. *The Principles of Nuclear Magnetism* (Oxford University Press, 1961).

[8] Carof, A., Salanne, M., Charpentier, T. & Rotenberg, B. On the microscopic fluctuations driving the NMR relaxation of quadrupolar ions in water. *J. Chem. Phys.* **143**, 194504 (2015).

[9] Abascal, J. L. F. & Vega, C. A general purpose model for the condensed phases of water: TIP4P/2005. *J. Chem. Phys.* **123**, 234505 (2005).

[10] Chubak, I., Scalfi, L., Carof, A. & Rotenberg, B. NMR relaxation rates of quadrupolar aqueous ions from classical molecular dynamics using force-field specific Sternheimer factors. *J. Chem. Theor. Comput.* **17**, 6006–6017 (2021).

[11] Pitzer, K. S., Peiper, J. C. & Busey, R. H. Thermodynamic properties of aqueous sodium chloride solutions. *J. Phys. Chem. Ref. Data* **13**, 1–102 (1984).

[12] Marin-Laflèche, A. *et al.* Metalwalls: A classical molecular dynamics software dedicated to the simulation of electrochemical systems. *J. Open Source Softw.* **5**, 2373 (2020).

[13] Nosé, S. A unified formulation of the constant temperature molecular dynamics methods. *J. Chem. Phys.* **81**, 511–519 (1984).

[14] Hoover, W. G. Canonical dynamics: Equilibrium phase-space distributions. *Phys. Rev. A* **31**, 1695–1697 (1985).

[15] Martyna, G. J., Tobias, D. J. & Klein, M. L. Constant pressure molecular dynamics algorithms. *J. Chem. Phys.* **101**, 4177–4189 (1994).

[16] Singer, P. M. *et al.* Predicting $^1$H NMR relaxation in $Gd^{3+}$-aqua using molecular dynamics simulations. *Phys. Chem. Chem. Phys.* **23**, 20974–20984 (2021).

[17] Honegger, P. *et al.* Understanding the nature of nuclear magnetic resonance relaxation by means of fast-field-cycling relaxometry and molecular dynamics simulations—the validity of relaxation models. *J. Phys. Chem. Lett.* **11**, 2165–2170 (2020).

[18] Singer, P. M., Asthagiri, D., Chapman, W. G. & Hirasaki, G. J. Molecular dynamics simulations of NMR relaxation and diffusion of bulk hydrocarbons and water. *J. Magn. Reson.* **277**, 15–24 (2017).

[19] Bosse, J., Quitmann, D. & Wetzel, C. Mode-coupling theory of field-gradient correlation functions: The quadrupolar relaxation rate in liquids. *Phys. Rev. A* **28**, 2459–2473 (1983).

[20] Asta, A. J., Levesque, M., Vuilleumier, R. & Rotenberg, B. Transient hydrodynamic finite-size effects in simulations under periodic boundary conditions. *Phys. Rev. E* **95**, 061301 (2017).

[21] Zhang, C., Yue, S., Panagiotopoulos, A. Z., Klein, M. L. & Wu, X. Dissolving salt is not equivalent to applying a pressure on water. *Nat. Commun.* **13**, 822 (2022).

[22] Sun, J., Ruzsinszky, A. & Perdew, J. P. Strongly constrained and appropriately normed semilocal density functional. *Phys. Rev. Lett.* **115**, 036402 (2015).

[23] Versmold, H. Interaction induced magnetic relaxation of quadrupolar ionic nuclei in electrolyte solutions. *Mol. Phys.* **57**, 201–216 (1986).

[24] Weingärtner, H. The mechanism of $^7$Li relaxation in a supercooled aqueous LiI solution. *J. Magn. Reson.* **41**, 74–87 (1980).

[25] Mohammadi, M., Benders, S. & Jerschow, A. Nuclear magnetic resonance spin-lattice relaxation of lithium ions in aqueous solution by NMR and molecular dynamics. *J. Chem. Phys.* **153**, 184502 (2020).

[26] Zhou, L., Xu, J., Xu, L. & Wu, X. Importance of Van der Waals effects on the hydration of metal ions from the Hofmeister series. *J. Chem. Phys.* **150**, 124505 (2019).

[27] Berberan-Santos, M., Bodunov, E. & Valeur, B. Mathematical functions for the analysis of luminescence decays with underlying distributions 1. Kohlrausch decay function (stretched exponential). *Chem. Phys.* **315**, 171–182 (2005).
